# Upregulation of Leukemia Inhibitory Factor (LIF) during the Early Stage of Optic Nerve Regeneration in Zebrafish

**DOI:** 10.1371/journal.pone.0106010

**Published:** 2014-08-27

**Authors:** Kazuhiro Ogai, Ayaka Kuwana, Suguru Hisano, Mikiko Nagashima, Yoshiki Koriyama, Kayo Sugitani, Kazuhiro Mawatari, Hiroshi Nakashima, Satoru Kato

**Affiliations:** 1 Department of Molecular Neurobiology, Graduate School of Medical Science, Kanazawa University, Kanazawa, Ishikawa, Japan; 2 Wellness Promotion Science Center, Institute of Medical, Pharmaceutical and Health Sciences, Kanazawa University, Kanazawa, Ishikawa, Japan; 3 Department of Clinical Laboratory Science, Graduate School of Medical Science, Kanazawa University, Kanazawa, Ishikawa, Japan; 4 Graduate School and Faculty of Pharmaceutical Sciences, Suzuka University of Medical Science, Suzuka, Mie, Japan; NIH/NEI, United States of America

## Abstract

Fish retinal ganglion cells (RGCs) can regenerate their axons after optic nerve injury, whereas mammalian RGCs normally fail to do so. Interleukin 6 (IL-6)-type cytokines are involved in cell differentiation, proliferation, survival, and axon regrowth; thus, they may play a role in the regeneration of zebrafish RGCs after injury. In this study, we assessed the expression of IL-6-type cytokines and found that one of them, leukemia inhibitory factor (LIF), is upregulated in zebrafish RGCs at 3 days post-injury (dpi). We then demonstrated the activation of signal transducer and activator of transcription 3 (STAT3), a downstream target of LIF, at 3–5 dpi. To determine the function of LIF, we performed a LIF knockdown experiment using LIF-specific antisense morpholino oligonucleotides (LIF MOs). LIF MOs, which were introduced into zebrafish RGCs via a severed optic nerve, reduced the expression of LIF and abrogated the activation of STAT3 in RGCs after injury. These results suggest that upregulated LIF drives Janus kinase (Jak)/STAT3 signaling in zebrafish RGCs after nerve injury. In addition, the LIF knockdown impaired axon sprouting in retinal explant culture *in* *vitro*; reduced the expression of a regeneration-associated molecule, growth-associated protein 43 (GAP-43); and delayed functional recovery after optic nerve injury *in* *vivo*. In this study, we comprehensively demonstrate the beneficial role of LIF in optic nerve regeneration and functional recovery in adult zebrafish.

## Introduction

Fish retinal ganglion cells (RGCs) are able to survive and regenerate their axons even after transection of the optic nerve, whereas mammalian RGCs cannot survive after injury [1], [2]. Accordingly, a number of studies have searched for the factors that enable fish RGCs to survive and regrow their axons after optic nerve injury (for review, see [3], [4]). Identifying and demonstrating the functions of such regeneration-associated genes, especially at the early stage of fish optic nerve regeneration, might offer new and reliable strategies for facilitating mammalian optic nerve regeneration [5], [6].

Interleukin 6 (IL-6)-type cytokines, such as IL-6, ciliary neurotrophic factor (CNTF), oncostatin M (OSM), cardiotrophin-1 (CT-1), and leukemia inhibitory factor (LIF), perform important and versatile functions in intercellular communication via membrane receptors and the Janus kinase (Jak)/signal transducer and activator of transcription 3 (STAT3) signaling pathway. Secreted IL-6-type cytokines first bind to the cell surface receptor gp130 or a cytokine-specific receptor, and this event triggers the phosphorylation of the Jak protein adjacent to the receptors. The phosphorylated Jak subsequently phosphorylates the STAT3 protein, which in turn forms a homodimer and works as a transcription factor [7]–[9].

Among the IL-6-type cytokines, IL-6, CNTF, and LIF have been described as “injury factors”, produced in response to damage of the peripheral and sympathetic nervous systems, which show regenerative capacity (for review, see [10]). These data imply that IL-6-type cytokines are regeneration-associated molecules. In fact, studies on IL-6, CNTF [11]–[14], LIF [15], and the activation of STAT3 after optic nerve injury in zebrafish [16] have suggested that these molecules have axon-regenerative properties in the central nervous system. At present, however, there are no reports in the literature that are focused specifically on the expression, localization, and function of these cytokines during zebrafish optic nerve regeneration.

In this study, we assess the possible influence of the intrinsic expression of IL-6-type cytokines, especially IL-6, CNTF, and LIF on the regeneration of zebrafish retina after optic nerve injury. Because among the three IL-6-type cytokines, only LIF shows significant upregulation after injury, we studied the expression and function of LIF in detail as the main regeneration-associated molecule. Here we show the upregulation of LIF and subsequent activation of STAT3 at the early stage of zebrafish optic nerve regeneration. We then identify the function of the upregulated LIF and activated STAT3 in nerve regeneration by the means of an LIF knockdown with a behavioral test *in* *vivo*. We also demonstrate the relationship between the LIF upregulation and growth-associated protein 43 (GAP-43) expression. This link may point to the function of LIF as a “switch” for other regenerative pathways during optic nerve regeneration in zebrafish.

## Materials and Methods

### Ethics statement

All experimental procedures were approved by the Committee on Animal Experimentation of Kanazawa University (#86294), and care was taken to minimize pain and the number of fish used. All surgery was performed under anesthesia by ethyl 3-aminobenzoate methanesulfonic acid (MS222), which is commonly used for fish anesthesia. For sacrifice of fish, we employed a general method in which fish were killed by an overdose (0.1%) of MS222 for 5 min.

### Animals and the surgical procedure

Adult zebrafish (*Danio rerio*, 3–4 cm long; between the ages of 6 and 12 months) were used throughout this study. Optic nerve injury was caused according to the method of Münzel et al. [17] with a slight modification. In brief, fish were anesthetized by immersion in 0.02% MS222 (Sigma–Aldrich, MO, USA) in 10 mM PBS (pH 7.4). Under anesthesia, the optic nerve was carefully severed 1 mm posterior to the eyeball using microscissors. The fish were reared in 28°C water until appropriate time points.

### Total RNA extraction and cDNA synthesis

At appropriate time points after the optic nerve injury, fish were killed by an overdose (0.1%) of MS222/PBS (5 min). Retinas were then excised and stored at −80°C until use. For total RNA extraction, we used Sepasol-RNA I Super G (Nacalai Tesque, Kyoto, Japan) or Nucleospin RNA extraction kit (Takara, Shiga, Japan) according to the manufacturer’s instructions. Total RNA samples from each time point or treatment were subjected to first-strand cDNA synthesis using ReverTra Ace qPCR RT Master Mix with the gDNA Remover kit (Toyobo, Osaka, Japan).

### PCRs

We performed quantitative real-time PCR to measure the mRNA expression of IL-6, CNTF, and LIF using the Thunderbird SYBR qPCR Mix (Toyobo) and gene-specific primers (Table S1) with the Mx3005P qPCR System (Agilent Technologies, CA, USA) according to the manufacturer’s instructions. The expression level was analyzed using the ΔΔCt method [18] after we confirmed PCR efficiency of each primer pair. β-Actin was used as a reference gene. We performed qualitative PCR using the SapphireAmp Fast PCR Master Mix (Takara) and gene-specific primers (Table S2). PCR products were then analyzed by electrophoresis on an agarose gel, stained with ethidium bromide, and documented using the GeneGenius 2 Bio Imaging System (Syngene, MD, USA).

### Tissue preparation

At appropriate time points, the fish were killed and their eyeballs were removed, followed by fixation with 4% paraformaldehyde in PBS overnight at 4°C. The eyeballs were then immersed in 30% sucrose in PBS overnight at 4°C for cryoprotection and embedded in optimal cutting temperature compound (Sakura Finetek, Tokyo, Japan). The retinal cryosections (thickness 12 µm) were prepared and stored at −80°C until analysis.

### *In situ* hybridization

*In situ* hybridization was performed as described previously with slight modifications [19]. In brief, retinal slices were rehydrated and permeabilized using 5 µg/mL proteinase K (Life Technologies) at 25°C for 5 min. After acetylation and prehybridization, the slices were incubated with a digoxigenin (DIG)-labeled antisense complementary RNA (cRNA) probe overnight at 55°C. The next day, the excess probe was washed off, and the slices were incubated with an alkaline phosphatase (AP)-conjugated anti-DIG antibody (1∶500 dilution; Roche Applied Science, Mannheim, Germany) overnight at 4°C. On the third day, the slices were washed, and the positive signals were visualized by means of nitro blue tetrazolium (NBT)/5-bromo-4-chloro-3-indolyl phosphate (BCIP; Roche Applied Science). The slices incubated with a sense probe served as a negative control.

### Western blotting

Retinas were isolated in the same manner as total RNA and then sonicated in 50 µL of lysis buffer [15]. The supernatants were collected and the protein concentration was measured by means of the Bradford method (Bio-Rad, CA, USA). Western blotting was performed as described elsewhere [20]. In brief, 20 µg of total protein from each time point or treatment was subjected to (9% or 15%) polyacrylamide gel electrophoresis, and the separated proteins were transferred to a nitrocellulose membrane (Whatman, Maidstone, UK). The membrane was washed, blocked, and incubated with a primary antibody (Table S3) overnight at 4°C. Then, the membrane was washed and incubated with an appropriate AP- or horseradish peroxidase (HRP)-conjugated secondary antibody (1∶1000; Sigma–Aldrich or Santa Cruz, respectively) for 1 hour at 25°C followed by visualization of the protein bands using the BCIP/NBT Phosphatase Substrate System (KPL, MD, USA) or Clarity Western ECL Substrate (Bio-Rad), respectively. Densitometric analysis was performed using the ImageJ software. β-Actin served as a loading control.

### Immunohistochemical analysis

The retinal slices were incubated with 10 mM citrate buffer (pH 6.0) for 5 min at 121°C for antigen retrieval. After washing, the slices were blocked and incubated with primary antibodies (Table S3) overnight at 4°C. After another washing step, the slices were incubated with the appropriate Alexa Fluor 488- or 594-conjugated secondary antibody (1∶500; Life Technologies) for 1 hour at 25°C, followed by counter-staining with 2 µg/mL 4′,6-diamidino-2-phenylindole (DAPI; Wako Pure Chemical Industries, Osaka, Japan). Immunoreactivity signals were photographed using a fluorescence microscope (VB-7000 or BZ-9000, Keyence, Osaka, Japan).

### Application of the morpholino oligonucleotides

To downregulate the expression of zebrafish LIF *in* *vivo* after optic nerve injury, we prepared the following two zebrafish LIF-targeting morpholino oligonucleotides (MOs): MO(1), 5′-AGT GTG GCG GTA ATA CTT ACT GAA T-3′, targeting the exon 1–intron 1 splice donor; MO(2), 5′-CAA TCT CTG AGA CAG GCA GAG CAT G-3′, targeting the initiation codon. A standard MO (5′-CCT CTT ACC TCA GTT ACA ATT TAT A-3′), whose sequence is absent from the zebrafish genome, was used as a control MO [21], [22]. All MOs were labeled with fluorescein isothiocyanate (FITC) and purchased from GeneTools, OR, USA. Retrograde transport of FITC-tagged MO into zebrafish RGCs was performed according to the method of Becker et al. [23] and Veldman et al. [21]. In brief, a small piece of gelatin foam (Spongel; Astellas, Tokyo, Japan) was soaked with 1 µL of 1 mM MO and was applied to the cut site of the severed optic nerve immediately after the nerve injury. In this method, MOs were neither tagged with Vivo-Porter [24], [25] nor coadministered with Endo-Porter [26]. Therefore, the effects of MOs on nonneuronal cells (e.g., astrocytes or oligodendrocytes), in the optic nerve adjacent to the MO application site, can be considered negligible.

### Retinal explant culture

Retinal explant culture was set up according to the method of Sugitani et al. [19]. In brief, retinas were aseptically isolated and cut into approximately 0.5 mm×0.5 mm squares. The retinal explants were then cultured in the Leibovitz L-15 medium (Life Technologies) supplemented with 20 mM HEPES (pH 7.2; Sigma–Aldrich), 10% fetal bovine serum (Life Technologies), and antibiotics (Wako Pure Chemical Industries) in a poly-L-lysine (Sigma–Aldrich)-coated dish at 28°C for 5 days. The percentage of explants showing neurite outgrowth was assessed using the ImageJ software.

### Behavioral experiments

To evaluate the recovery of visual function after optic nerve injury, the optomotor response (OMR) was tested using the equipment designed by our group [27] based on the findings of Neuhauss et al. [28]. In brief, fish were placed into an annular water tank surrounded by rotating black and white stripes. Fish will swim randomly when the stripes are not moving. On the other hand, when the stripes are rotating, fish with an intact visual system (or restored enough to recognize the stripes) will chase the rotating stripes. In our experiments, the stripes were rotated at 72°/sec clockwise and counterclockwise. The fish movements were captured by a video camera placed 1 m above the tank, and the swimming direction of fish was analyzed using a custom-designed software [22], [27]. The concordance ratio was then calculated as follows [27]:
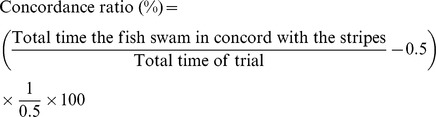



In this calculation, the concordance ratio ranges from 0% (completely random swimming) to 100% (completely concordant swimming).

### Statistical analysis

The expression levels of LIF mRNA, LIF protein, phospho-STAT3 (pSTAT3), and GAP-43 were calculated as mean ± SEM, and the differences were evaluated using one-way analysis of variance (ANOVA) followed by Tukey’s *post hoc* test. The levels of active caspase-3 and Bcl-2 were calculated as mean ± SEM and were analyzed using Student’s *t*-test. We calculated the percentage of explants showing neurite outgrowth (>100 µm long) and used the chi-square test followed by Bonferroni’s *post hoc* test to analyze intergroup differences. To evaluate the results of the behavioral test, two-way ANOVA followed by Tukey’s *post hoc* test was used. In all cases, calculations were performed using the Prism software (version 6.0c; GraphPad Software, CA, USA), and a *p* value<0.05 was considered statistically significant.

## Results

### Upregulation of LIF mRNA and protein in RGCs after optic nerve injury in adult zebrafish

**Figure 1:**
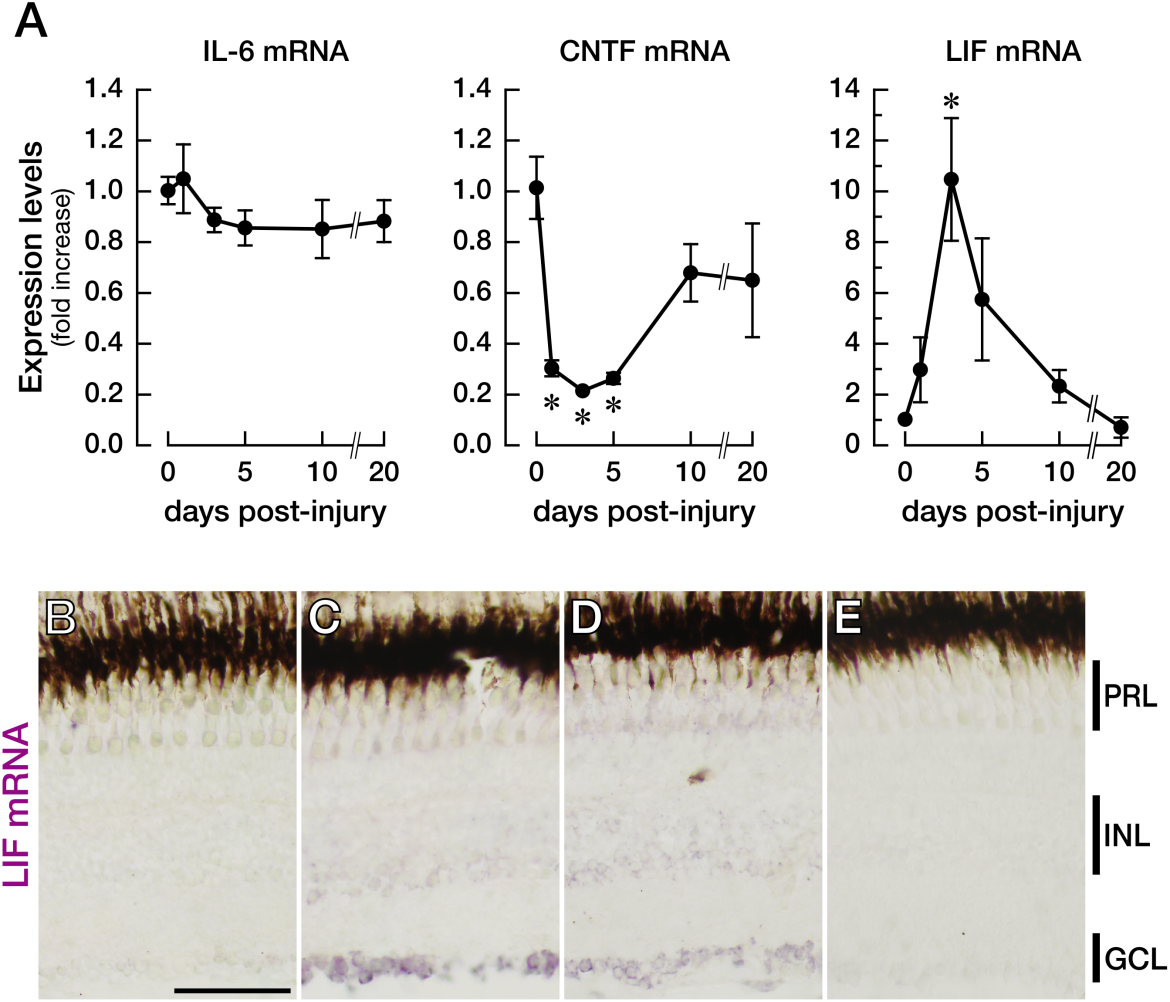
Upregulation of LIF mRNA in zebrafish RGCs after optic nerve injury. (A) IL-6 mRNA showed little or no change for at least 20 days after the nerve injury, but CNTF mRNA showed significant downregulation 1–5 days post-injury (dpi; *n* = 3, *p<0.01). The expression level of LIF mRNA drastically increased at 3 dpi (*n* = 3, *p<0.01). (B–E) *In situ* hybridization revealed very weak expression of LIF mRNA in the intact zebrafish retina (B). At 3 and 5 dpi, LIF mRNA was upregulated mostly in the ganglion cell layer [GCL; weakly in the inner nuclear layer (INL) and photoreceptor layer (PRL)] (C and D, respectively). Incubation with the sense probe yielded no signal even at 3 dpi (E). The scale bar in (B) is 50 µm.

We first measured changes in the expression of three IL-6-type cytokines (IL-6, CNTF, and LIF) in the zebrafish retina after optic nerve injury. IL-6 mRNA expression showed little or no change for at least 20 days post-injury (dpi). The level of CNTF mRNA showed a significant decrease at 1–5 dpi. In contrast, LIF mRNA levels increased 10.5-fold (*n* = 3; *p* = 0.004) at 3 dpi and returned to baseline by 10–20 dpi (Fig. 1A). To determine the localization of LIF mRNA in the zebrafish retina and to confirm the quantitative PCR result, we performed *in* *situ* hybridization using an LIF-specific antisense cRNA probe. In an intact retina, there was a weak expression of LIF mRNA in the ganglion cell layer (GCL; Fig. 1B). The expression of LIF mRNA became stronger mostly in GCL at 3 dpi (Fig. 1C) and became weaker at 5 dpi (Fig. 1D), consistent with the results of quantitative PCR (Fig. 1A). A very weak positive signal of LIF mRNA was also observed in the lower part of the inner nuclear layer (INL) and the photoreceptor layer (PRL). *In situ* hybridization using a sense probe yielded no positive signals even at 3 dpi (Fig. 1E).

**Figure 2:**
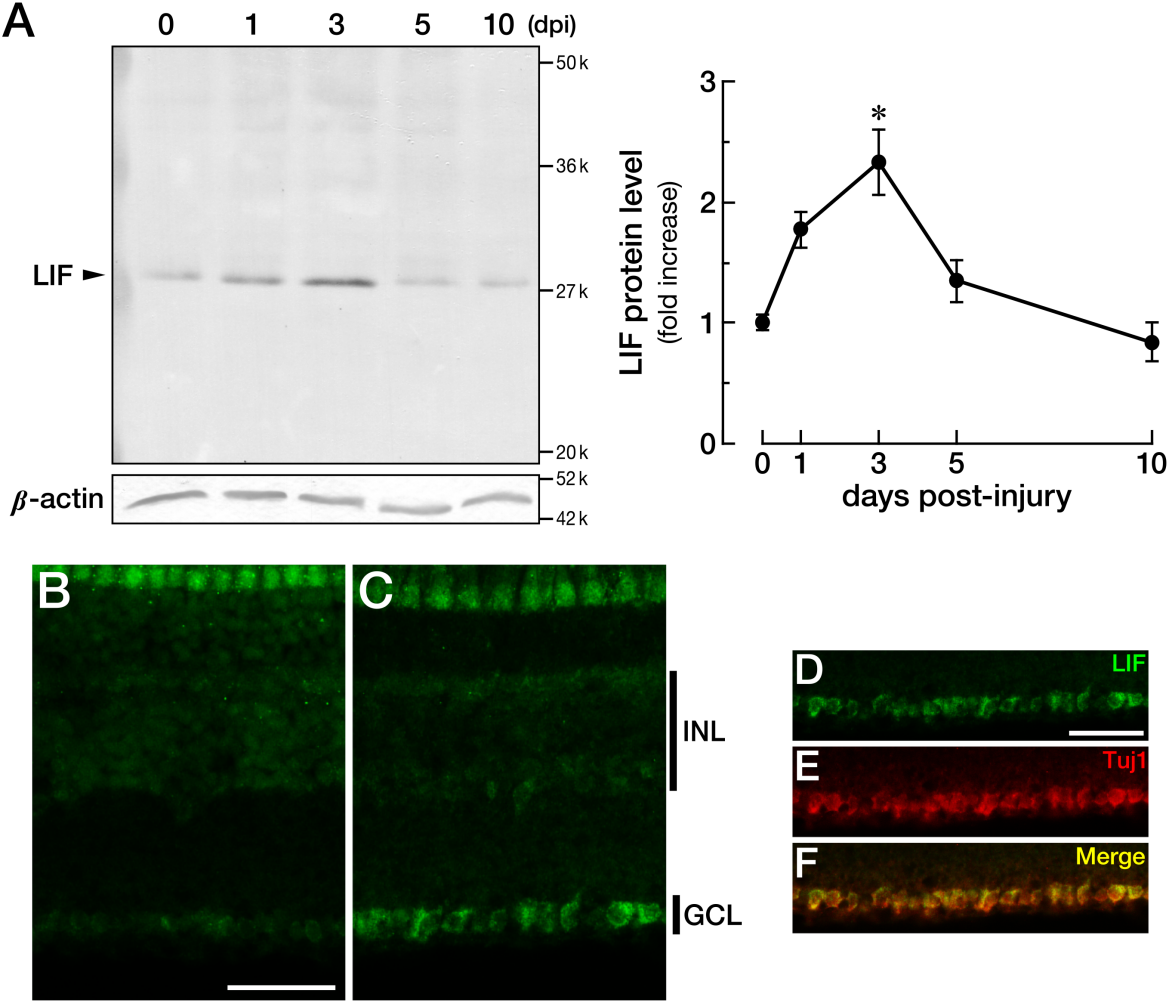
Upregulation of the LIF protein in zebrafish RGCs after optic nerve injury. (A) As with the expression changes of LIF mRNA, the LIF protein was also upregulated starting on day 1 after the optic nerve injury, peaked at 3 days, and returned to baseline by 5 days post-injury (*n* = 3, *p<0.001). (B, C) Immunohistochemical analysis of the LIF protein expression revealed that LIF was upregulated mainly in GCL at 3 dpi (C) compared with the intact retina (B). (D–F) Double immunohistochemical staining of LIF (D) and Tuj1 (E) revealed that LIF is produced in RGCs (F). The scale bars in (B) and (D) are 30 µm.

We also examined the expression and localization of the LIF protein in the retina after injury (Fig. 2). As with LIF mRNA, LIF protein levels increased 2.3-fold (*n* = 3; *p* = 0.0003) at 3 dpi and returned to baseline by 5–10 dpi (Fig. 2A). The upregulation of the LIF protein was almost limited to GCL, as confirmed by immunohistochemical analysis of the LIF protein (control, Fig. 2B; 3 dpi, Fig. 2C). Very small upregulation of LIF in the innermost layer of INL was also observed; this result was consistent with the slight upregulation of LIF mRNA in INL, as shown in Fig. 1C. The pattern of immunoreactivity of LIF in GCL (Fig. 2C, D) completely matched that of Tuj1 (βIII tubulin; Fig. 2E, F), a marker of RGCs [19], [29]. This finding confirms that LIF was indeed produced in RGCs.

### Phosphorylation (activation) of STAT3 in the zebrafish retina after optic nerve injury

**Figure 3:**
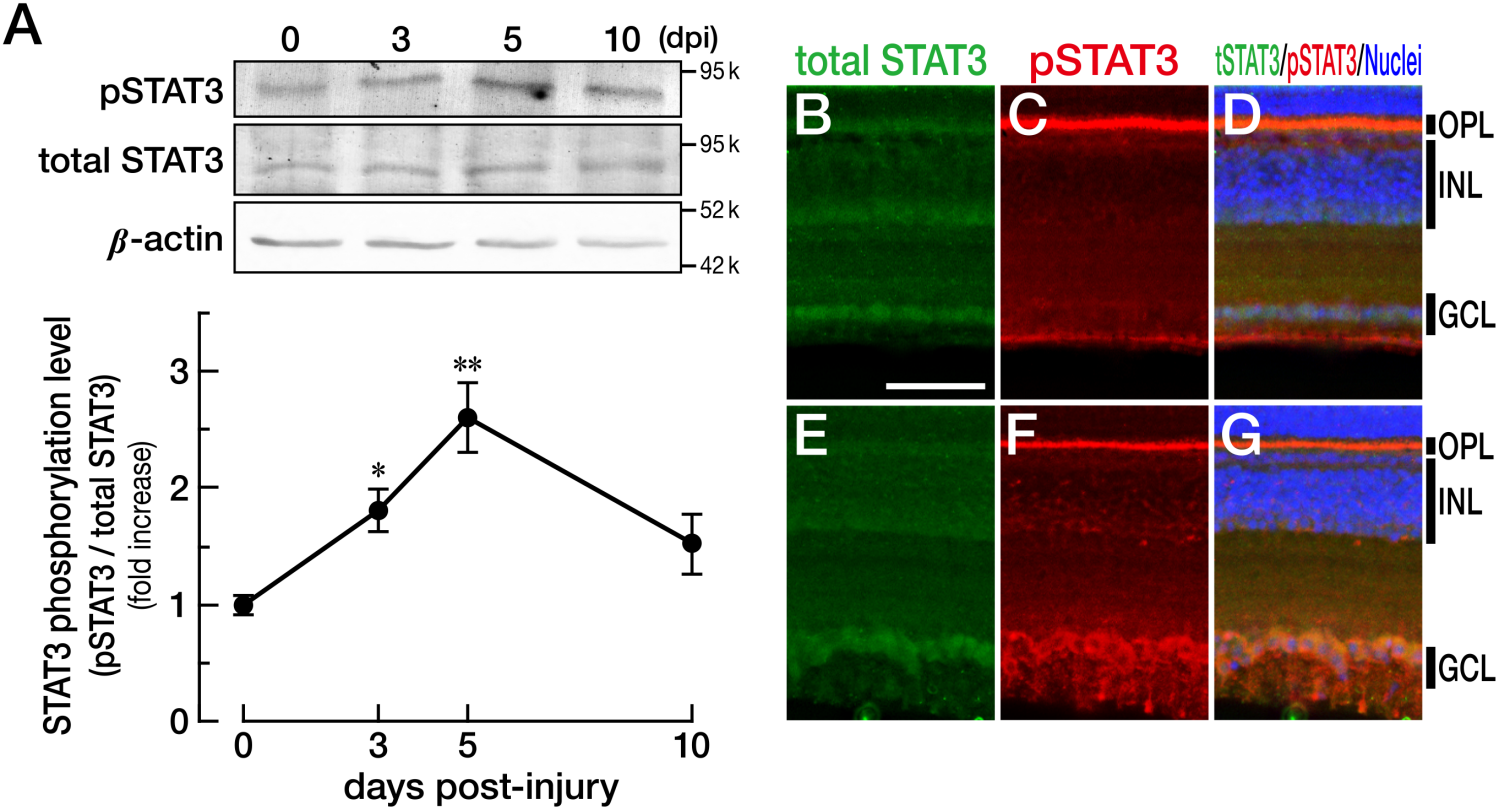
Activation of STAT3 in zebrafish RGCs after optic nerve injury. STAT3 is activated when its Tyr^708^ is phosphorylated (pSTAT3). (A) Western blotting of pSTAT3 revealed the activation of STAT3 at 3–5 dpi (*n* = 3, *p<0.05, **p<0.001). The total amount of STAT3 was not changed. (B–G) Immunohistochemical staining of total STAT3 and pSTAT3. Immunoreactivity corresponding to pSTAT3 in RGCs was drastically increased at 5 dpi (F) compared to the intact retina (C), whereas total STAT3 remained unchanged (B: intact, E: 5 dpi). (D) and (G) show merged images of (B, C) and (E, F), respectively, with nuclear staining for orientation. The scale bar in (B) is 50 µm. OPL: outer plexiform layer.

LIF triggers the phosphorylation of STAT3 via LIF receptor and Jak kinases, and pSTAT3 works as a transcription factor [7], [15]. Because LIF was upregulated in RGCs after optic nerve injury, we examined STAT3 phosphorylation (activation) in RGCs after the injury. Western blotting of pSTAT3 and total STAT3 (phosphorylated and unphosphorylated STAT3) showed that the amount of pSTAT3 relative to total STAT3 increased 1.8-fold (*n* = 3; *p* = 0.0319) and 2.6-fold (*n* = 3; *p* = 0.0007) at 3 and 5 dpi, respectively (Fig. 3A). The amount of total STAT3 did not change during this period. Immunohistochemical analysis revealed that in the intact retina, total STAT3 was predominantly present in GCL and the inner part of INL (Fig. 3B). In contrast, pSTAT3 was mainly detected in the outer plexiform layer (OPL) and was faintly detectable in the inner part of the retina (Fig. 3C, D). At 5 dpi, the expression pattern of total STAT3 did not change much (Fig. 3E). Nonetheless, as with western blotting, the positive signals of pSTAT3 drastically increased mainly in GCL (Fig. 3F, G).

### The knockdown of LIF attenuated the activation of STAT3

**Figure 4:**
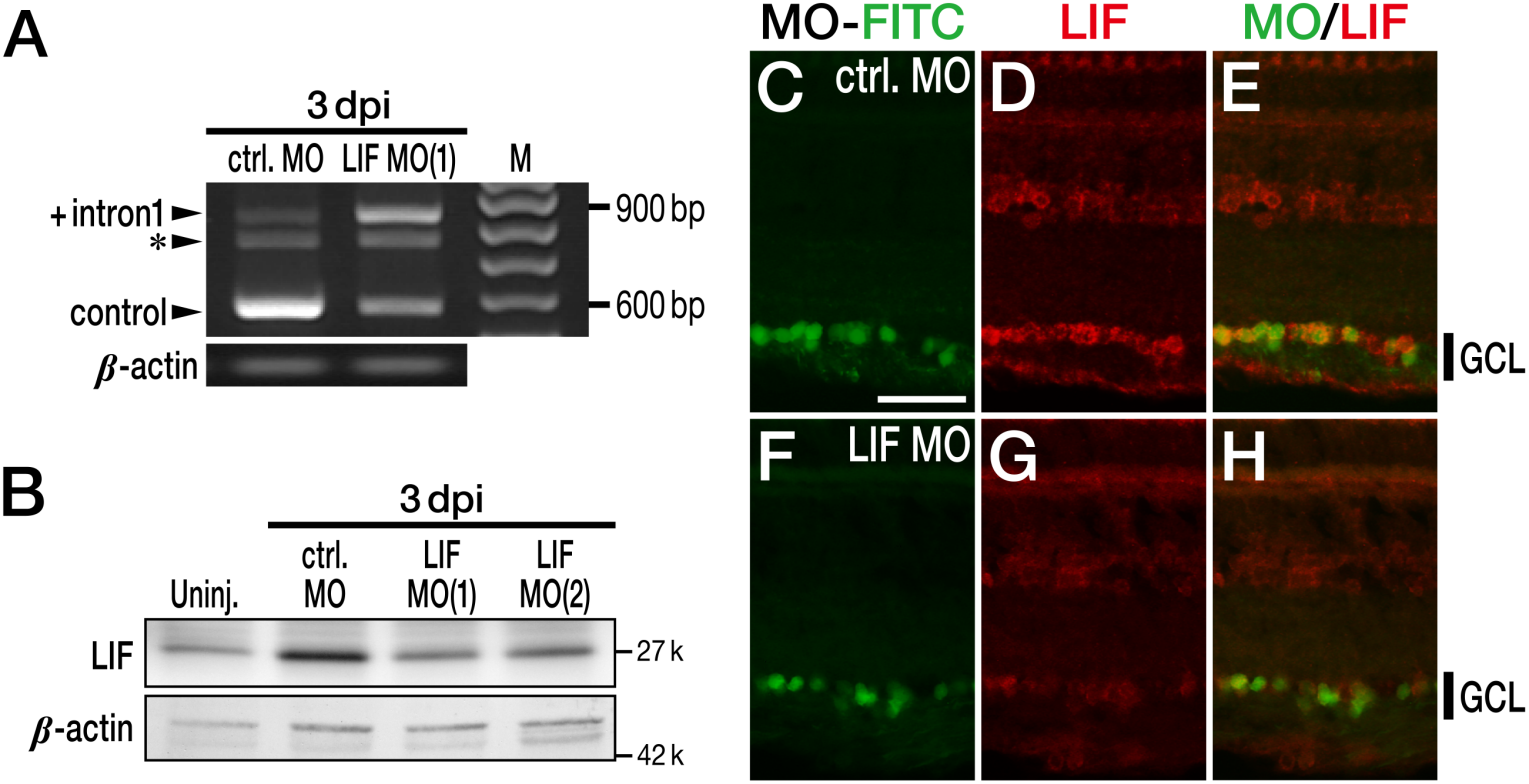
Knockdown of LIF in zebrafish RGCs after optic nerve injury. (A) Effects of LIF morpholino (MO) on the expression of LIF mRNA at 3 dpi. LIF MO(1) decreased the amount of control (i.e., normal) LIF mRNA product (control; 571 bp) and increased the amount of LIF with intron 1 (+intron1; 833 bp). The asterisk denotes a non-specific product. (B) Effects of LIF MO on the expression of LIF protein at 3 dpi. As expected, LIF MO(2), along with LIF MO(1), decreased the amount of LIF protein compared with the control (ctrl.) MO treatment. (C–H) Immunohistochemical analysis of LIF after MO treatment at 3 dpi. All MOs are labeled with FITC. LIF MO clearly attenuated the expression of LIF in the RGCs (F–H) compared with the treatment with control MO (C–E). The scale bar in (C) is 30 µm.

After optic nerve injury, upregulation of LIF takes place followed by STAT3 phosphorylation in RGCs. To prove the link between these two events, we applied LIF-targeting MOs to RGCs through the stump of the severed optic nerve. Two types of MOs were prepared. The first type, LIF MO(1), blocks the exon 1–intron 1 splice donor and causes the inclusion of intron 1, thereby producing a premature termination codon. The second MO is LIF MO(2), which blocks the initiation codon leading to the suppression of LIF protein translation. At 3 dpi, control (i.e., normal) LIF was downregulated and LIF with intron 1 (+intron1 LIF) was upregulated in the LIF MO(1)-treated retina compared with the control MO-treated retina (Fig. 4A). This result indicates that LIF MO(1) successfully stimulated the inclusion of intron 1. LIF protein expression was reduced in the LIF MO(2)-treated retina compared with the control MO-treated retina. LIF MO(1) also reduced the amount of LIF protein (Fig. 4B). Thus, both LIF MO(1) and LIF MO(2) reduced LIF gene and protein expression *in* *vivo*. The knockdown of the LIF protein by the means of LIF MOs (a cocktail of two MOs) was also confirmed by immunohistochemical staining of LIF. MOs were tagged with FITC so that they could be visualized using their green fluorescence. The upregulation of LIF in RGCs was detected in the control MO-treated retina at 3 dpi (Fig. 4C–E), whereas LIF expression was eliminated in the LIF MO-treated retina (Fig. 4F–H).

**Figure 5:**
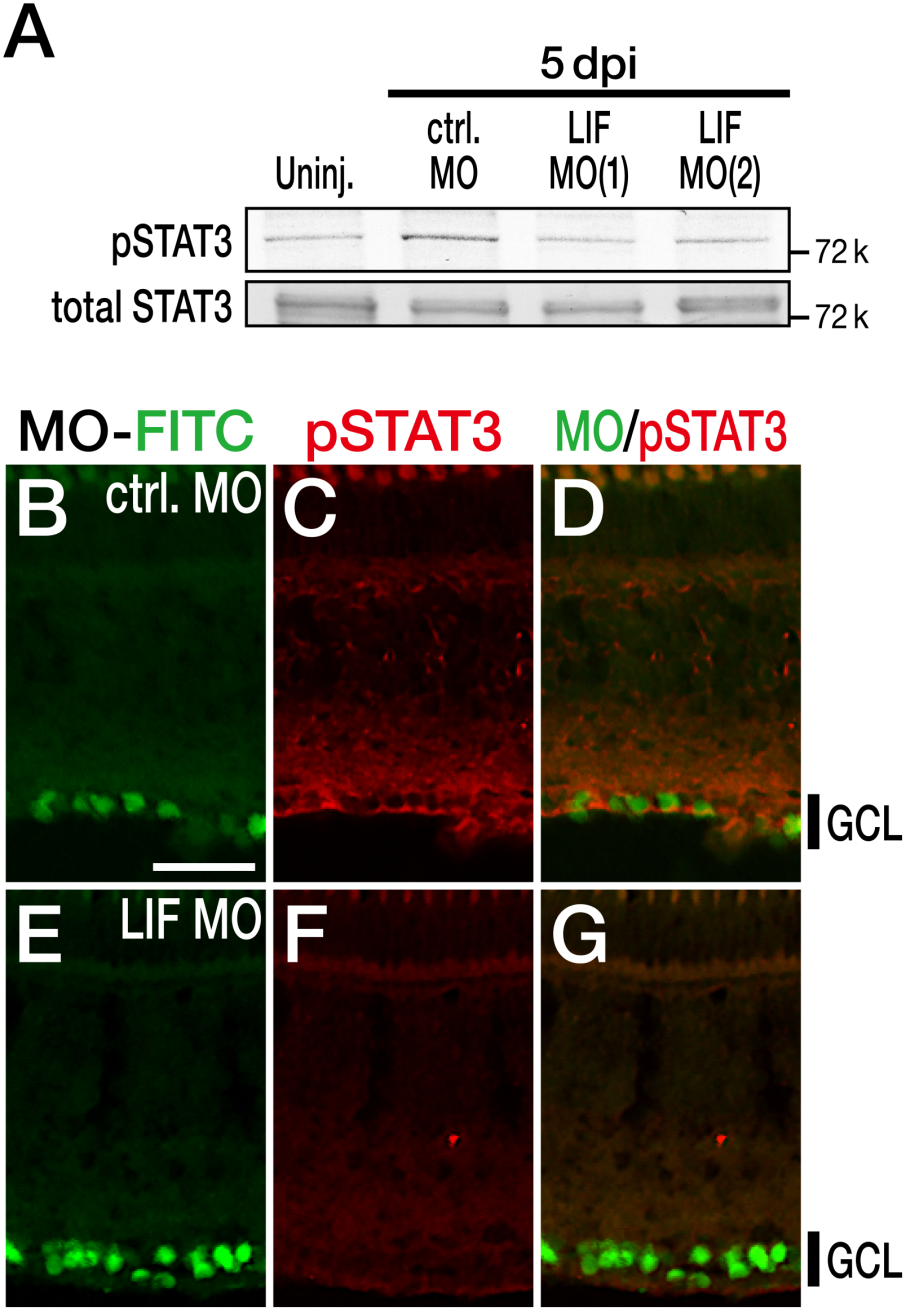
Decrease in the phosphorylation level of STAT3 after treatment with LIF MO. (A) The activation of STAT3 was attenuated by LIF MO, whereas the level total STAT3 protein was not affected at 5 dpi. (B–G) Immunohistochemical analysis of pSTAT3 after LIF MO treatment at 5 dpi. As with the LIF expression demonstrated in Fig. 4, LIF MO suppressed the activation of STAT3 in RGCs (E–G) compared with control MO treatment (B–D). The scale bar in (B) is 30 µm.

Next we tested the level of pSTAT3 after treatment with LIF MOs. The level of pSTAT3 increased in the control MO-treated retina at 5 dpi, whereas this level decreased in both LIF MO(1)- and LIF MO(2)-treated retinas (Fig. 5A). The level of total STAT3 was not affected by MO treatment. Immunohistochemical analysis of pSTAT3 also confirmed the decrease in the level of pSTAT3 in the LIF MO-treated retina (Fig. 5E–G) compared with the control MO-treated retina (Fig. 5B–D).

Taken together, these results confirmed that the knockdown of LIF in zebrafish RGCs after optic nerve injury attenuated the activation of STAT3, indicating that there was a connection between LIF and Jak/STAT3 signaling.

### The knockdown of LIF impaired axon regeneration and delayed recovery of visual function after optic nerve injury

Because LIF was upregulated at 3 days after optic nerve injury, when RGCs are preparing for axonal regrowth [4], we next explored the effects of LIF on axon regeneration and visual function recovery.

**Figure 6:**
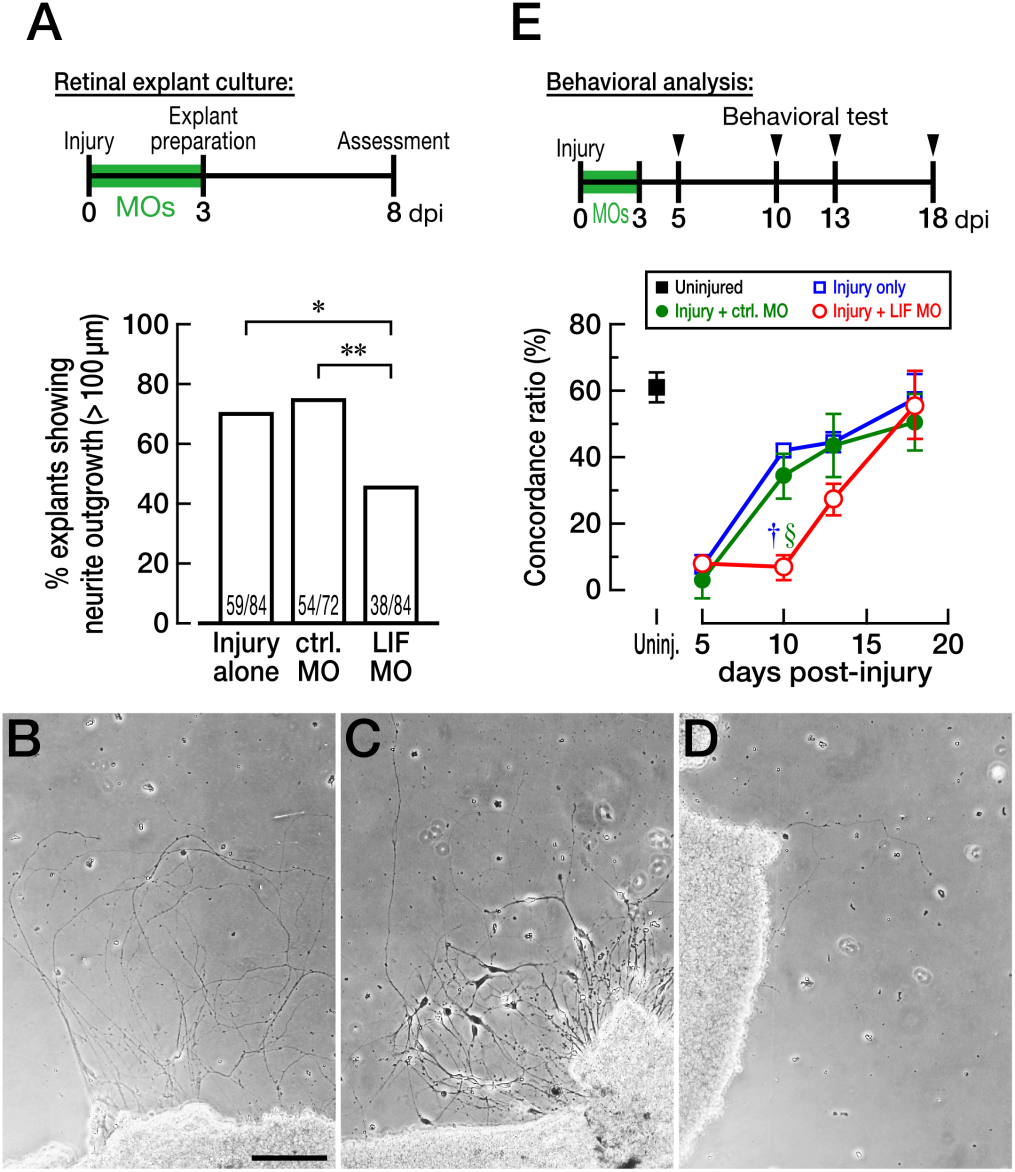
Impairment of axonal regrowth and functional recovery by the LIF knockdown after optic nerve injury. (A) The experiment with retinal explant culture. The LIF knockdown reduced the percentage of explants showing neurite outgrowth (>100 µm) compared with optic nerve injury alone and control LIF MO-treated retina. The chi-square test was used to assess the significance (*p<0.01 vs. injury alone; **p<0.001 vs control MO treatment). (B–D) Representative images of retinal explants. (B) injury alone, (C) treated with control MO, and (D) treated with LIF MO. (E) Behavioral analysis with or without MO treatment. LIF MO significantly delayed functional recovery of OMR at 10 dpi (*n* = 6, †*p*<0.05 vs. injury alone; *n* = 6, §*p*<0.05 vs. control MO treatment) and comparable recovery was observed at 18 dpi. The scale bar in (B) is 100 µm. Uninj.: uninjured.

First, we prepared a retinal explant culture where a control MO or LIF MO were introduced beforehand. Retinal explants treated with MOs were prepared at 3 dpi and cultured for another 5 days (Fig. 6A). The percentage of explants showing neurite outgrowth significantly decreased in LIF MO-treated explants compared with injury-alone explants or control MO-treated ones (Fig. 6A; *p* = 0.004 and *p* = 0.0007, respectively). Representative images of retinal explants with injury alone, control MO, and LIF MO were shown in Figure 6B, C, and D, respectively.

Second, we tested the recovery of visual function after LIF knockdown in RGCs using the OMR test [27] in fish treated with a control MO or LIF MO at 5, 10, 13, and 18 dpi (Fig. 6E). In this experiment, fish swim in an annular water tank surrounded by rotating black and white stripes. If the visual function of fish is intact or restored, then the fish chase the stripes; thus, the direction of the moving stripes and the fish should be the same (“concord”). On the other hand, if the visual function is not working well enough to recognize the moving stripes, then the affected fish swim randomly (i.e., sometimes in concord and sometimes in discord). To quantify the swimming performance, we expressed the concord between the stripes and swimming as a concordance ratio (Fig. 6E; [27]). The uninjured fish showed approximately 60% concordance ratio, and the ratio drastically dropped to <10% at 5 dpi in all groups. At 10 dpi, fish with the optic nerve injury alone and those treated with control MO showed considerable recovery to approximately 40% of the normal. Nevertheless, fish treated with LIF MO showed no recovery (*n* = 6, *p* = 0.0002 vs injury-alone group and *p* = 0.0092 vs control MO-treated group, at 10 dpi). The fish treated with LIF MO then showed gradual recovery, and the concordance ratio became comparable (approximately 50%) to that in the uninjured fish by 20 dpi.

Taken together, these data showed that LIF may have a beneficial effect on the onset, but not on the later stages, of the regenerative process after optic nerve injury.

### The knockdown of LIF reduced the expression level of GAP-43

**Figure 7:**
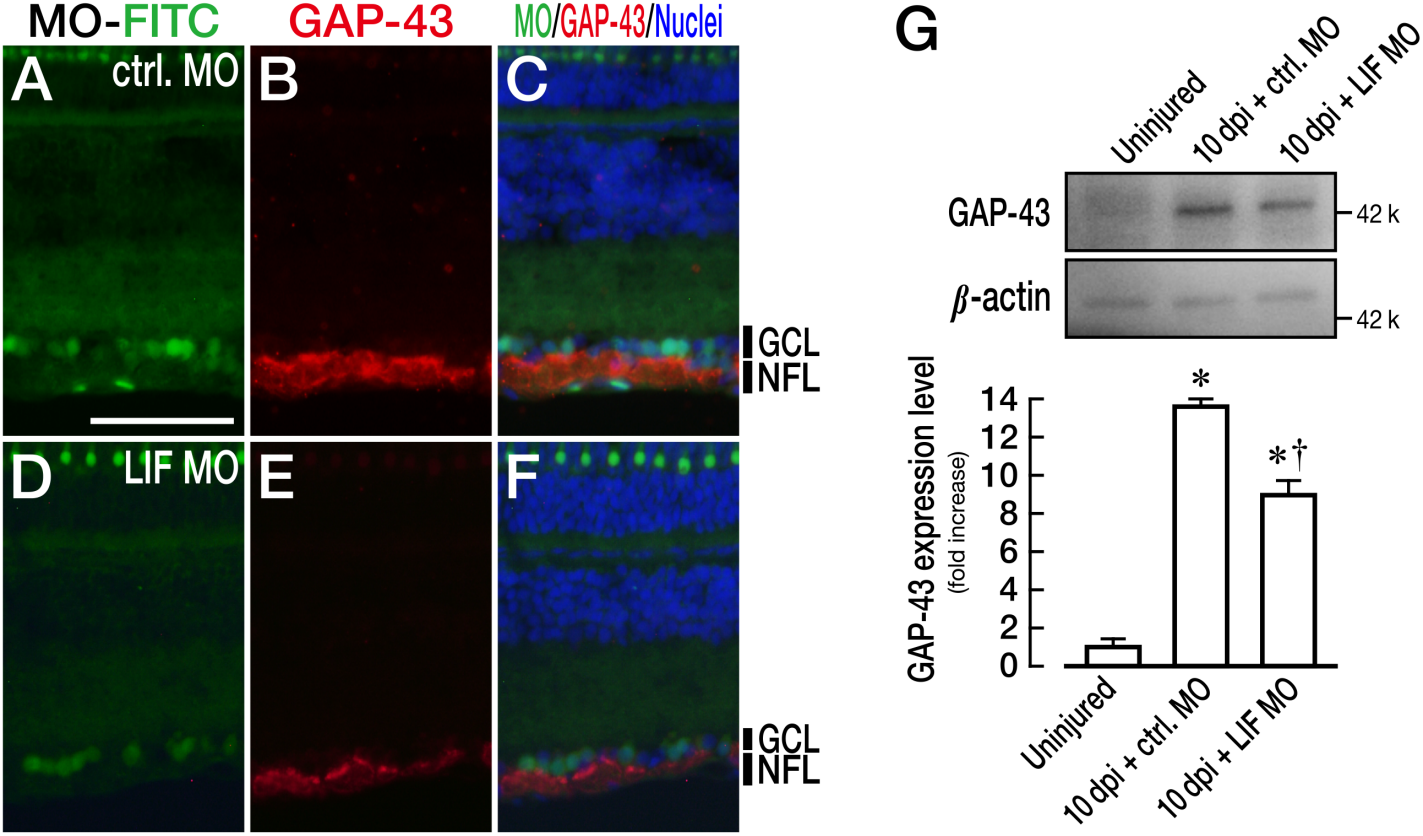
Reduction in GAP-43 expression by an LIF knockdown. (A–F) Immunohistochemical analysis of GAP-43 in the MO-treated retina at 5 dpi. LIF MO reduced the expression of GAP-43 (D–F) compared with control MO treatment (A–C) in the nerve fiber layer (NFL). (G) Quantification of GAP-43 expression by western blotting. GAP-43 was significantly upregulated at 10 dpi (*n* = 4, *p<0.001 vs. uninjured). LIF MO significantly reduced the expression level of GAP-43 compared with control MO treatment (*n* = 4, †*p*<0.001 vs. control MO). The scale bar in (A) is 50 µm.

Next, we performed immunohistochemical analysis of GAP-43, which is one of the regeneration-associated molecules during zebrafish optic nerve regeneration [27], [30]–[32], because the expression of GAP-43 is associated with STAT3 activation [33]. The immunohistochemical and western blot analyses of the GAP-43 protein were performed at 10 dpi when functional recovery was significantly different between control MO-treated and LIF MO-treated fish (Fig. 6E). As shown in Figure 7A–F, LIF MO clearly attenuated the expression of GAP-43 in nerve fiber layer (NFL; Fig. 7D–F) compared with the control MO-treated retina (Fig. 7A–C). Quantification of the level of GAP-43 protein in the retina confirmed the downregulation of GAP-43 in the LIF MO-treated retina compared with the control MO treatment (Fig. 7G; *n* = 4, *p* = 0.0008). These results indicate that the expression of LIF could trigger the expression of GAP-43 after optic nerve injury in adult zebrafish.

## Discussion

**Figure 8:**
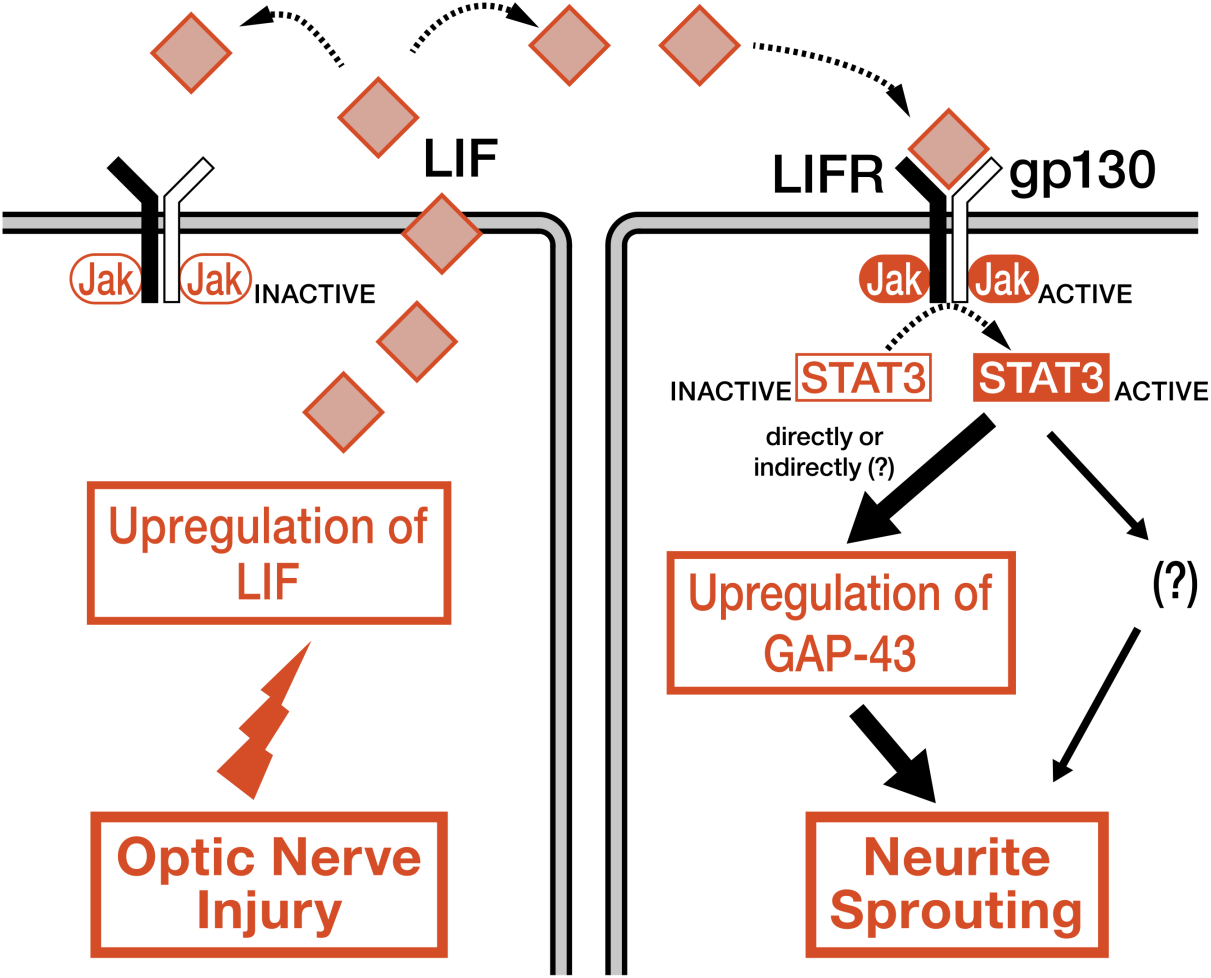
Schematic summary of the results. Zebrafish RGCs upregulate LIF in response to optic nerve injury. The resultant LIF then activates Jak/STAT3 signaling via LIF receptors in neighboring RGCs [or on the same (secreting) cell]. Activation of STAT3 leads to the upregulation of regeneration-associated molecules (e.g., GAP-43) directly or indirectly, which facilitates neurite sprouting and, consequently, optic nerve regeneration in adult zebrafish.

This study shows the intrinsic upregulation of LIF and activation of STAT3 in zebrafish RGCs after optic nerve injury. It also demonstrates that LIF is beneficial for regeneration of the optic nerve and for functional recovery via induction of GAP-43. The proposed mechanism of LIF/Jak/STAT3 signaling in zebrafish RGCs is shown in Figure 8.

### Upregulation of LIF at the early stage of zebrafish optic nerve regeneration

Unlike their mammalian counterparts, fish RGCs can survive and regrow their axons after optic nerve injury through the various proteins encoded by the so-called regeneration-associated genes [3], [4]. In this study, we focus on IL-6-type cytokines as candidates for regeneration-associated genes in the zebrafish retina after optic nerve injury because these cytokines are known to be expressed in injured neurons, for example, in the peripheral nervous system, which can regenerate its axons [10].

We chose three IL-6-type cytokines IL-6, CNTF, and LIF because these three factors are known as “injury factors” that are induced after peripheral nerve injury and have been studied regarding their effects on CNS regeneration [12]–[16], [34], [35]. We found that RGCs intrinsically upregulate LIF at 3 days after optic nerve injury (Fig. 1, 2), whereas IL-6 expression remains unchanged for at least 20 dpi (Fig. 1A). The process of optic nerve regeneration in adult zebrafish can be classified into four periods: preparation (1–4 dpi), axon elongation (5–30 dpi), synaptic refinement in the tectum (35–80 dpi), and full functional recovery (100–120 dpi) [4]. The time point of LIF upregulation (3 dpi) corresponds to the preparation stage of optic nerve regeneration, when injured RGCs assume a regenerative state such that they can produce new axons. The knockdown of LIF clearly impairs neurite sprouting and delays functional recovery after the injury (Fig. 6). Therefore, it can be speculated that LIF is beneficial, if not essential, for the sprouting of new axons after optic nerve injury in zebrafish.

We found it interesting that the level of CNTF mRNA significantly decreased 1–5 days after the nerve injury (Fig. 1A). There are far fewer studies on downregulated genes than on upregulated genes [21], and there are extremely few studies (to the best of our knowledge, at the moment, there is only one study) on zebrafish CNTF gene expression [36]. In addition, we did not analyze the localization of CNTF mRNA in the zebrafish retina. Therefore, it is difficult to predict the function of CNTF in nerve regeneration at this point. Given that the level of CNTF mRNA also decreases during heart regeneration, whereas other IL-6-type cytokines (including LIF) are upregulated [36], it seems that the intrinsic upregulation of CNTF might not be required for the regenerative process, although exogenous injection of CNTF is beneficial for photoreceptor survival after light-induced injury [37].

It is reported that there is constitutive neurogenesis at the edge of the fish retina, called ciliary marginal zone (CMZ) [38]. Therefore, it is possible that LIF is expressed in this region because LIF is also linked to cell proliferation. Nonetheless, at a lower magnification of LIF immunohistochemical images, we found that the LIF protein is mainly expressed in GCL, but not in CMZ, at 3 dpi (Fig. S1). For this reason, LIF may be a regeneration-specific molecule rather than a regulator of cell proliferation.

### Activation of Jak/STAT3 signaling after zebrafish optic nerve injury

LIF has a signal peptide targeting it to be secreted from the cell [39], and the secreted LIF affects its target cells via cell membrane receptors (LIF receptor and gp130) and via the intracellular Jak/STAT3 signaling pathway [7]. It has been reported that LIF receptor and gp130 are expressed in the eye of the larval zebrafish [40], [41]. The present study also confirms the expression of LIF receptors (LIFR-A and LIFR-B) and gp130 in RGCs of adult zebrafish (Fig. S2). Thus, zebrafish RGCs are ready to transduce LIF signals via those receptors.

We next demonstrated, in line with the findings of Elsaeidi et al. [16], that optic nerve injury causes the activation of STAT3 at 3–5 dpi, the stage that corresponds to upregulation of LIF (3 dpi; Figs. 1–3). We also show that a knockdown of LIF abrogates STAT3 activation (Fig. 5). The activation of STAT3 (3–5 dpi; Fig. 3A) is slightly lagging behind the peak of LIF expression (3 dpi; Fig. 2A). This slight difference could be explained by the secretion delay of the LIF protein. LIF affects its target cells via cell surface receptors; that is, in order to exert its action, LIF needs to be secreted from the cell. A study of blood mononuclear cells showed that the maximal secretion of IL-6 takes place 24 hours after lipopolysaccharide treatment [42]. Although the cell type and the cytokine are different here, we can hypothesize that the peak of LIF expression and that of pSTAT3 activation differ because of the time necessary for the secretion of LIF.

### The role of LIF and subsequent Jak/STAT3 signaling in zebrafish optic nerve regeneration

Previous studies have shown LIF and the Jak/STAT3 pathway to be axon regeneration promoters: LIF can stimulate neurite outgrowth from dorsal root ganglion neurons [43]; pSTAT3 acts as a retrograde signaling protein that promotes sensory and motor neuron regeneration [44]; STAT3 activation evoked by peripheral branch injury of the dorsal root ganglion promotes spinal axon regeneration [33]; in addition, STAT3 activation is required for zebrafish optic nerve regeneration according to the latest study [16]. Some investigators have confirmed that STAT3 activation acts to augment the expression of regeneration-associated molecules such as GAP-43 and small proline-rich protein 1a (SPRR1A) [14], [45], [46]. GAP-43 is known as a marker of growing and regrowing axons [30] and is indeed upregulated in RGCs during zebrafish optic nerve regeneration [27]. SPRR1A is reported to be expressed in axotomized neurons and promotes axon outgrowth [47]. Nonetheless, we were unable to find such a gene in the zebrafish genome so far. In this study, we show that a knockdown of LIF suppresses the expression of GAP-43 (Fig. 7) and impairs neurite outgrowth and functional recovery (Fig. 6). The GAP-43 protein, however, is not completely eliminated by LIF knockdown. Furthermore, the restoration of visual function is achieved, albeit with a delay, even after LIF knockdown. The GAP-43 gene expression is regulated not only by Jak/STAT3 signaling, but also by the extracellular signal-regulated kinase 1/2 and phosphatidylinositol 3-kinase (PI3K)/Akt pathway [48]. The latter is indeed activated during fish optic nerve regeneration [49]. Therefore, the intrinsic upregulation of LIF is one of the necessary events for regulating the expression of regeneration-associated genes (e.g., GAP-43), which are essential for neurite regrowth.

It has also been reported that Jak/STAT3 signaling is linked to the regulation of cell survival. STAT3 activation directly induces anti-apoptotic genes, Bcl-2 and Bcl-xL, in the neuronal cell line PC12 [50]. In a model of focal ischemic brain injury, the cells most positive for active caspase-3 staining (these cells will undergo apoptotic cell death) are pSTAT3 negative, and *vice versa*. These observations point to the STAT3 activation as a neuroprotective event capable of preventing ischemic cell death [51]. Given that fish RGCs can survive after nerve injury owing to the upregulation of Bcl-2 and suppression of caspase-3 activity [49], we can speculate that LIF and Jak/STAT3 signaling in zebrafish RGCs can also be crucial for cell survival after injury. However, neither Bcl-2 nor active caspase-3 levels are affected by the knockdown of LIF (Fig. S3). Because we could not directly detect apoptotic cell death (e.g., TUNEL assay), we cannot rule out the possibility of caspase-3-independent and Bcl-2-independent apoptosis. Nonetheless, we can theorize that after injury, LIF and STAT3 activation are not involved in cell survival, at least in caspase-3-dependent apoptotic mechanisms.

### Can LIF also be effective at regenerating a mammalian optic nerve?

We show here that LIF triggers axonal regrowth through GAP-43 induction after optic nerve injury in adult zebrafish. Because there is a report that LIF secreted from astrocytes ameliorates optic nerve regeneration under inflammatory conditions in mammals [15], therapeutic use of LIF for mammalian optic nerve regeneration is a possibility. However, some issues need to be resolved first. Lens injury, which triggers an inflammatory reaction in the eye, is reported to activate astrocytes adjacent to RGCs and to cause astrocytes to produce LIF (and CNTF), which in turn promotes axon growth in the mouse retina [15]. On the other hand, another study shows that lens injury is not related to either astrocyte activation or LIF/CNTF production but rather to macrophage infiltration and production of oncomodulin (which may be responsible for axon regeneration just like LIF and CNTF) [52]–[54]. These two arguments are open to debate. In addition, LIF shows quite low homology in its amino acid sequence among species: homology is only 14.9% between humans and zebrafish. There are some investigators who tried to produce a species-specific LIF to maintain species-specific stem cells (i.e., rat LIF for rat stem cells, chicken LIF for chicken stem cells, and so on) [55]–[57]. In our preliminary experiments, indeed, the addition of recombinant *mouse* LIF cannot induce neurite sprouting in *zebrafish* retinal explant culture (Fig. S4; *p* = 0.76, the chi-square test). The function of fish LIF is similar to that of mammalian LIF to the extent that LIF induces myeloid cell differentiation [58] and affects nerve regeneration [15]. Thus, we can hypothesize that the binding affinity of LIF for LIF receptor varies among species because of the variation in the amino acid sequence. Due to the above problems, it is difficult to say whether LIF can be used directly for mammalian optic nerve regeneration. Nonetheless, the proposed LIF/Jak/STAT3/GAP-43 cascade may be a promising source of molecules that can be used therapeutically for mammalian optic nerve regeneration. First, the function of LIF in nerve regeneration needs to be confirmed in mammals, and species-specific LIF needs to be prepared and tested.

In conclusion, we have demonstrated the beneficial effects of LIF during the early stage of optic nerve regeneration in zebrafish. Accordingly, we believe that LIF, not IL-6 or CNTF, participates in the intrinsic regeneration process and in recovery of visual function in adult zebrafish after optic nerve injury. The proposed notion that the LIF/Jak/STAT3/GAP-43 pathway is involved in axonal regrowth may lead to the identification of new therapeutic targets in optic nerve diseases.

## Supporting Information

Figure S1 **Expression of LIF in the ciliary marginal zone.** Immunohistochemical staining of LIF on a retinal slice (at 3 dpi) revealed that LIF was strongly expressed in the ganglion cell layer (GCL; solid arrowheads), but not in the ciliary marginal zone (CMZ; open arrowheads). The scale bar is 100 µm. INL: inner nuclear layer, ONL: outer nuclear layer.
(TIF)

Figure S2 **LIF receptor expression in the retina of adult zebrafish.** (A) RT-PCR of two LIF receptors (LIFR-A and LIFR-B) and gp130 on uninjured retinal cells. All of these receptor genes are expressed in the zebrafish retina, although their expression levels vary greatly. (B–G) *In situ* hybridization experiments with LIF receptors. The localization of LIFR-A, LIFR-B, and gp130 mRNA is shown in (B), (C), and (D), respectively. All of the receptors are expressed in the GCL and other cell layers at the same levels as shown by RT-PCR (A). Sense probes yielded no specific staining (E–G). The scale bar in (G) is 50 µm. GCL: ganglion cell layer, INL: inner nuclear layer, PRL: photoreceptor layer.
(TIF)

Figure S3 **No changes in the expression of pro-apoptotic and anti-apoptotic factors after a LIF knockdown.** (A–C) Immunohistochemical staining of active caspase-3, the effector molecule of apoptotic cell death, in LIF MO-treated retina at 10 dpi. Caspase-3 was not activated by LIF knockdown. (D) Changes in Bcl-2, the anti-apoptotic factor, and active caspase-3 levels in the control MO- and LIF MO-treated retina (10 dpi). Neither the level of Bcl-2 nor that of active caspase-3 was affected by LIF knockdown (*n* = 4; *p* = 0.88 and *p* = 0.89, respectively); n.s.: not significant. GCL: ganglion cell layer.
(TIF)

Figure S4 **Addition of recombinant mouse LIF to zebrafish retinal explant culture.** Supplementation with recombinant mouse LIF had no effect on the ratio of explants showing neurite outgrowth (*p* = 0.76); n.s.: not significant.
(TIF)

Table S1 **Primers used for quantitative real-time PCR.**
(PDF)

Table S2 **Primers used for qualitative PCR and in** **situ hybridization probe.**
(PDF)

Table S3 **Primary antibodies used in this study.**
(PDF)

## References

[1] Attardi DG, Sperry RW (1963) Preferential selection of central pathways by regenerating optic fibers. Exp Neurol 7: 46–64. 13965388 10.1016/0014-4886(63)90093-1

[2] Berkelaar M, Clarke DB, Wang YC, Bray GM, Aguayo AJ (1994) Axotomy results in delayed death and apoptosis of retinal ganglion cells in adult rats. J Neurosci 14: 4368–4374. 8027784 10.1523/JNEUROSCI.14-07-04368.1994 PMC6577016

[3] Becker CG, Becker T (2007) Growth and pathfinding of regenerating axons in the optic projection of adult fish. J Neurosci Res 85: 2793–2799. 17131420 10.1002/jnr.21121

[4] Kato S, Matsukawa T, Koriyama Y, Sugitani K, Ogai K (2013) A molecular mechanism of optic nerve regeneration in fish: the retinoid signaling pathway. Prog Retin Eye Res 37: 13–30. 23994437 10.1016/j.preteyeres.2013.07.004

[5] Sugitani K, Matsukawa T, Koriyama Y, Shintani T, Nakamura T, et al (2006) Upregulation of retinal transglutaminase during the axonal elongation stage of goldfish optic nerve regeneration. Neuroscience 142: 1081–1092. 16997488 10.1016/j.neuroscience.2006.07.042

[6] Homma K, Koriyama Y, Mawatari K, Higuchi Y, Kosaka J, et al (2007) Early downregulation of IGF-I decides the fate of rat retinal ganglion cells after optic nerve injury. Neurochem Int 50: 741–748. 17363111 10.1016/j.neuint.2007.01.011

[7] Heinrich PC, Behrmann I, Haan S, Hermanns HM, Müller-Newen G, et al (2003) Principles of interleukin (IL)-6-type cytokine signalling and its regulation. Biochem J 374: 1–20. 12773095 10.1042/BJ20030407 PMC1223585

[8] Heinrich PC, Behrmann I, Müller-Newen G, Schaper F, Graeve L (1998) Interleukin-6-type cytokine signalling through the gp130/Jak/STAT pathway. Biochem J 334 (Pt 2): 297–314. 10.1042/bj3340297 PMC1219691 9716487

[9] Aaronson DS, Horvath CM (2002) A road map for those who don’t know JAK-STAT. Science 296: 1653–1655. 12040185 10.1126/science.1071545

[10] Zigmond RE (2011) gp130 cytokines are positive signals triggering changes in gene expression and axon outgrowth in peripheral neurons following injury. Front Mol Neurosci 4: 62. 22319466 10.3389/fnmol.2011.00062 PMC3262188

[11] Park K, Luo JM, Hisheh S, Harvey AR, Cui Q (2004) Cellular mechanisms associated with spontaneous and ciliary neurotrophic factor-cAMP-induced survival and axonal regeneration of adult retinal ganglion cells. J Neurosci 24: 10806–10815. 15574731 10.1523/JNEUROSCI.3532-04.2004 PMC6730205

[12] Müller A, Hauk TG, Fischer D (2007) Astrocyte-derived CNTF switches mature RGCs to a regenerative state following inflammatory stimulation. Brain 130: 3308–3320. 17971355 10.1093/brain/awm257

[13] Yang P, Wen H, Ou S, Cui J, Fan D (2012) IL-6 promotes regeneration and functional recovery after cortical spinal tract injury by reactivating intrinsic growth program of neurons and enhancing synapse formation. Exp Neurol 236: 19–27. 22504113 10.1016/j.expneurol.2012.03.019

[14] Pernet V, Joly S, Jordi N, Dalkara D, Guzik-Kornacka A, et al (2013) Misguidance and modulation of axonal regeneration by Stat3 and Rho/ROCK signaling in the transparent optic nerve. Cell Death Dis 4: e734. 23868067 10.1038/cddis.2013.266 PMC3730436

[15] Leibinger M, Müller A, Andreadaki A, Hauk TG, Kirsch M, et al (2009) Neuroprotective and axon growth-promoting effects following inflammatory stimulation on mature retinal ganglion cells in mice depend on ciliary neurotrophic factor and leukemia inhibitory factor. J Neurosci 29: 14334–14341. 19906980 10.1523/JNEUROSCI.2770-09.2009 PMC6665071

[16] Elsaeidi F, Bemben MA, Zhao XF, Goldman D (2014) Jak/STAT signaling stimulates zebrafish optic nerve regeneration and overcomes the inhibitory actions of socs3 and sfpq. J Neurosci 34: 2632–2644. 24523552 10.1523/JNEUROSCI.3898-13.2014 PMC3921430

[17] Münzel EJ, Schaefer K, Obirei B, Kremmer E, Burton EA, et al (2012) Claudin k is specifically expressed in cells that form myelin during development of the nervous system and regeneration of the optic nerve in adult zebrafish. Glia 60: 253–270. 22020875 10.1002/glia.21260

[18] Livak KJ, Schmittgen TD (2001) Analysis of relative gene expression data using real-time quantitative PCR and the 2(-Delta Delta C(T)) Method. Methods 25: 402–408. 11846609 10.1006/meth.2001.1262

[19] Sugitani K, Ogai K, Hitomi K, Nakamura-Yonehara K, Shintani T, et al (2012) A distinct effect of transient and sustained upregulation of cellular factor XIII in the goldfish retina and optic nerve on optic nerve regeneration. Neurochem Int 61: 423–432. 22709671 10.1016/j.neuint.2012.06.004

[20] Ogai K, Hisano S, Mawatari K, Sugitani K, Koriyama Y, et al (2012) Upregulation of anti-apoptotic factors in upper motor neurons after spinal cord injury in adult zebrafish. Neurochem Int 61: 1202–1211. 22982298 10.1016/j.neuint.2012.08.015

[21] Veldman MB, Bemben MA, Thompson RC, Goldman D (2007) Gene expression analysis of zebrafish retinal ganglion cells during optic nerve regeneration identifies KLF6a and KLF7a as important regulators of axon regeneration. Dev Biol 312: 596–612. 17949705 10.1016/j.ydbio.2007.09.019

[22] Nagashima M, Mawatari K, Tanaka M, Higashi T, Saito H, et al (2009) Purpurin is a key molecule for cell differentiation during the early development of zebrafish retina. Brain Res 1302: 54–63. 19748496 10.1016/j.brainres.2009.09.020

[23] Becker CG, Lieberoth BC, Morellini F, Feldner J, Becker T, et al (2004) L1.1 is involved in spinal cord regeneration in adult zebrafish. J Neurosci 24: 7837–7842. 15356195 10.1523/JNEUROSCI.2420-04.2004 PMC6729920

[24] Guo Y, Ma L, Cristofanilli M, Hart RP, Hao A, et al (2011) Transcription factor Sox11b is involved in spinal cord regeneration in adult zebrafish. Neuroscience 172: 329–341. 20951776 10.1016/j.neuroscience.2010.10.026 PMC3292217

[25] Lin JF, Pan HC, Ma LP, Shen YQ, Schachner M (2012) The cell neural adhesion molecule contactin-2 (TAG-1) is beneficial for functional recovery after spinal cord injury in adult zebrafish. PLoS One 7: e52376. 23285014 10.1371/journal.pone.0052376 PMC3528781

[26] Maxhimer JB, Soto-Pantoja DR, Ridnour LA, Shih HB, Degraff WG, et al (2009) Radioprotection in normal tissue and delayed tumor growth by blockade of CD47 signaling. Sci Transl Med 1: 3ra7. 10.1126/scitranslmed.3000139 PMC2811586 20161613

[27] Kaneda M, Nagashima M, Nunome T, Muramatsu T, Yamada Y, et al (2008) Changes of phospho-growth-associated protein 43 (phospho-GAP43) in the zebrafish retina after optic nerve injury: a long-term observation. Neurosci Res 61: 281–288. 18485507 10.1016/j.neures.2008.03.008

[28] Neuhauss SC, Biehlmaier O, Seeliger MW, Das T, Kohler K, et al (1999) Genetic disorders of vision revealed by a behavioral screen of 400 essential loci in zebrafish. J Neurosci 19: 8603–8615. 10493760 10.1523/JNEUROSCI.19-19-08603.1999 PMC6783047

[29] Fischer D, He Z, Benowitz LI (2004) Counteracting the Nogo receptor enhances optic nerve regeneration if retinal ganglion cells are in an active growth state. J Neurosci 24: 1646–1651. 14973241 10.1523/JNEUROSCI.5119-03.2004 PMC6730473

[30] Skene JH (1989) Axonal growth-associated proteins. Annu Rev Neurosci 12: 127–156. 2648946 10.1146/annurev.ne.12.030189.001015

[31] Benowitz LI, Routtenberg A (1997) GAP-43: an intrinsic determinant of neuronal development and plasticity. Trends Neurosci 20: 84–91. 9023877 10.1016/s0166-2236(96)10072-2

[32] Schaden H, Stuermer CA, Bähr M (1994) GAP-43 immunoreactivity and axon regeneration in retinal ganglion cells of the rat. J Neurobiol 25: 1570–1578. 7861120 10.1002/neu.480251209

[33] Qiu J, Cafferty WB, McMahon SB, Thompson SW (2005) Conditioning injury-induced spinal axon regeneration requires signal transducer and activator of transcription 3 activation. J Neurosci 25: 1645–1653. 15716400 10.1523/JNEUROSCI.3269-04.2005 PMC6725934

[34] Ip NY, Wiegand SJ, Morse J, Rudge JS (1993) Injury-induced regulation of ciliary neurotrophic factor mRNA in the adult rat brain. Eur J Neurosci 5: 25–33. 8261087 10.1111/j.1460-9568.1993.tb00201.x

[35] Leibinger M, Müller A, Gobrecht P, Diekmann H, Andreadaki A, et al (2013) Interleukin-6 contributes to CNS axon regeneration upon inflammatory stimulation. Cell Death Dis 4: e609. 23618907 10.1038/cddis.2013.126 PMC3641349

[36] Fang Y, Gupta V, Karra R, Holdway JE, Kikuchi K, et al (2013) Translational profiling of cardiomyocytes identifies an early Jak1/Stat3 injury response required for zebrafish heart regeneration. Proc Natl Acad Sci U S A 110: 13416–13421. 23901114 10.1073/pnas.1309810110 PMC3746860

[37] Kassen SC, Thummel R, Campochiaro LA, Harding MJ, Bennett NA, et al (2009) CNTF induces photoreceptor neuroprotection and Müller glial cell proliferation through two different signaling pathways in the adult zebrafish retina. Exp Eye Res 88: 1051–1064. 19450453 10.1016/j.exer.2009.01.007

[38] Raymond PA, Barthel LK, Bernardos RL, Perkowski JJ (2006) Molecular characterization of retinal stem cells and their niches in adult zebrafish. BMC Dev Biol 6: 36. 16872490 10.1186/1471-213X-6-36 PMC1564002

[39] Abe T, Mikekado T, Haga S, Kisara Y, Watanabe K, et al (2007) Identification, cDNA cloning, and mRNA localization of a zebrafish ortholog of leukemia inhibitory factor. Comp Biochem Physiol B Biochem Mol Biol 147: 38–44. 17307004 10.1016/j.cbpb.2006.12.019

[40] Hanington PC, Patten SA, Reaume LM, Waskiewicz AJ, Belosevic M, et al (2008) Analysis of leukemia inhibitory factor and leukemia inhibitory factor receptor in embryonic and adult zebrafish (Danio rerio). Dev Biol 314: 250–260. 18201692 10.1016/j.ydbio.2007.10.012

[41] Varela M, Dios S, Novoa B, Figueras A (2012) Characterisation, expression and ontogeny of interleukin-6 and its receptors in zebrafish (Danio rerio). Dev Comp Immunol 37: 97–106. 22107841 10.1016/j.dci.2011.11.004

[42] Schindler R, Mancilla J, Endres S, Ghorbani R, Clark SC, et al (1990) Correlations and interactions in the production of interleukin-6 (IL-6), IL-1, and tumor necrosis factor (TNF) in human blood mononuclear cells: IL-6 suppresses IL-1 and TNF. Blood 75: 40–47. 2294996

[43] Cafferty WB, Gardiner NJ, Gavazzi I, Powell J, McMahon SB, et al (2001) Leukemia inhibitory factor determines the growth status of injured adult sensory neurons. J Neurosci 21: 7161–7170. 11549727 10.1523/JNEUROSCI.21-18-07161.2001 PMC6762988

[44] Lee N, Neitzel KL, Devlin BK, MacLennan AJ (2004) STAT3 phosphorylation in injured axons before sensory and motor neuron nuclei: potential role for STAT3 as a retrograde signaling transcription factor. J Comp Neurol 474: 535–545. 15174071 10.1002/cne.20140

[45] Wu YY, Bradshaw RA (1996) Induction of neurite outgrowth by interleukin-6 is accompanied by activation of Stat3 signaling pathway in a variant PC12 cell (E2) line. J Biol Chem 271: 13023–13032. 8662645 10.1074/jbc.271.22.13023

[46] Pradervand S, Yasukawa H, Muller OG, Kjekshus H, Nakamura T, et al (2004) Small proline-rich protein 1A is a gp130 pathway- and stress-inducible cardioprotective protein. EMBO J 23: 4517–4525. 15510217 10.1038/sj.emboj.7600454 PMC526469

[47] Bonilla IE, Tanabe K, Strittmatter SM (2002) Small proline-rich repeat protein 1A is expressed by axotomized neurons and promotes axonal outgrowth. J Neurosci 22: 1303–1315. 11850458 10.1523/JNEUROSCI.22-04-01303.2002 PMC6757578

[48] Liu Z, Cai H, Zhang P, Li H, Liu H, et al (2012) Activation of ERK1/2 and PI3K/Akt by IGF-1 on GAP-43 expression in DRG neurons with excitotoxicity induced by glutamate in vitro. Cell Mol Neurobiol 32: 191–200. 21822733 10.1007/s10571-011-9746-6 PMC11498431

[49] Koriyama Y, Homma K, Sugitani K, Higuchi Y, Matsukawa T, et al (2007) Upregulation of IGF-I in the goldfish retinal ganglion cells during the early stage of optic nerve regeneration. Neurochem Int 50: 749–756. 17363112 10.1016/j.neuint.2007.01.012

[50] Stephanou A, Brar BK, Knight RA, Latchman DS (2000) Opposing actions of STAT-1 and STAT-3 on the Bcl-2 and Bcl-x promoters. Cell Death Differ 7: 329–330. 10866494 10.1038/sj.cdd.4400656

[51] Yamashita T, Sawamoto K, Suzuki S, Suzuki N, Adachi K, et al (2005) Blockade of interleukin-6 signaling aggravates ischemic cerebral damage in mice: possible involvement of Stat3 activation in the protection of neurons. J Neurochem 94: 459–468. 15998296 10.1111/j.1471-4159.2005.03227.x

[52] Leon S, Yin Y, Nguyen J, Irwin N, Benowitz LI (2000) Lens injury stimulates axon regeneration in the mature rat optic nerve. J Neurosci 20: 4615–4626. 10844031 10.1523/JNEUROSCI.20-12-04615.2000 PMC6772462

[53] Yin Y, Cui Q, Li Y, Irwin N, Fischer D, et al (2003) Macrophage-derived factors stimulate optic nerve regeneration. J Neurosci 23: 2284–2293. 12657687 10.1523/JNEUROSCI.23-06-02284.2003 PMC6742044

[54] Yin Y, Henzl MT, Lorber B, Nakazawa T, Thomas TT, et al (2006) Oncomodulin is a macrophage-derived signal for axon regeneration in retinal ganglion cells. Nat Neurosci 9: 843–852. 16699509 10.1038/nn1701

[55] Takahama Y, Ochiya T, Sasaki H, Baba-Toriyama H, Konishi H, et al (1998) Molecular cloning and functional analysis of cDNA encoding a rat leukemia inhibitory factor: towards generation of pluripotent rat embryonic stem cells. Oncogene 16: 3189–3196. 9671398 10.1038/sj.onc.1201826

[56] Horiuchi H, Tategaki A, Yamashita Y, Hisamatsu H, Ogawa M, et al (2004) Chicken leukemia inhibitory factor maintains chicken embryonic stem cells in the undifferentiated state. J Biol Chem 279: 24514–24520. 15044464 10.1074/jbc.M313231200

[57] Xue F, Ma Y, Chen YE, Zhang J, Lin TA, et al (2012) Recombinant rabbit leukemia inhibitory factor and rabbit embryonic fibroblasts support the derivation and maintenance of rabbit embryonic stem cells. Cell Reprogram 14: 364–376. 22775411 10.1089/cell.2012.0001 PMC3411342

[58] Hanington PC, Belosevic M (2007) Interleukin-6 family cytokine M17 induces differentiation and nitric oxide response of goldfish (Carassius auratus L.) macrophages. Dev Comp Immunol 31: 817–829. 17250891 10.1016/j.dci.2006.12.001

