# Supplementary figures and images for: Upregulation of Leukemia Inhibitory Factor (LIF) during the Early Stage of Optic Nerve Regeneration in Zebrafish
Source: PLoS One. 2014 Aug 27;9(8):e106010. doi: 10.1371/journal.pone.0106010 (PMC4146584; doi:10.1371/journal.pone.0106010)

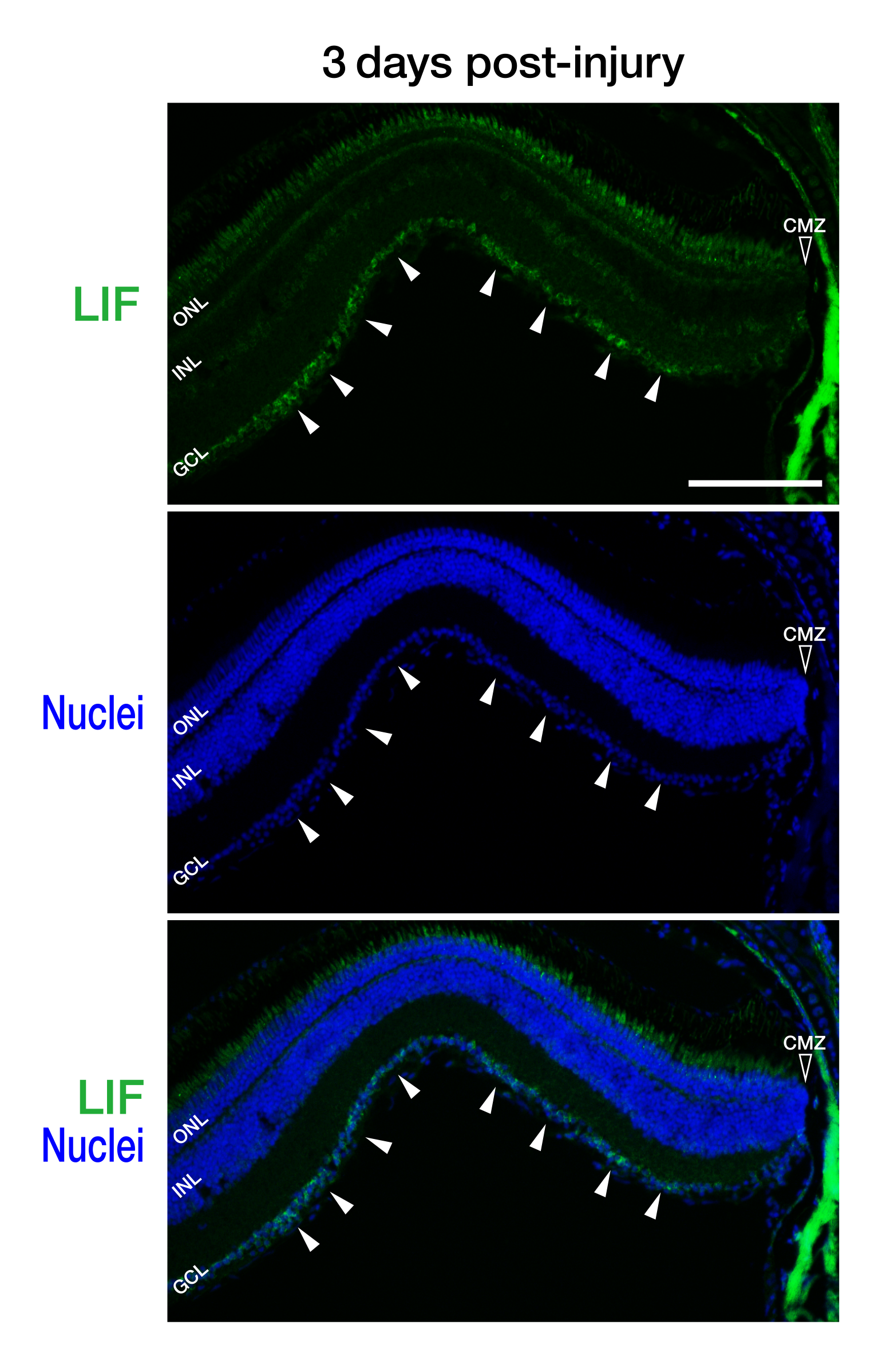

Supplement: Figure S1 — Expression of LIF in the ciliary marginal zone. Immunohistochemical staining of LIF on a retinal slice (at 3 dpi) revealed that LIF was strongly expressed in the ganglion cell layer (GCL; solid arrowheads), but not in the ciliary marginal zone (CMZ; open arrowheads). The scale bar is 100 µm. INL: inner nuclear layer, ONL: outer nuclear layer. (TIF) [file pone.0106010.s001.tif]

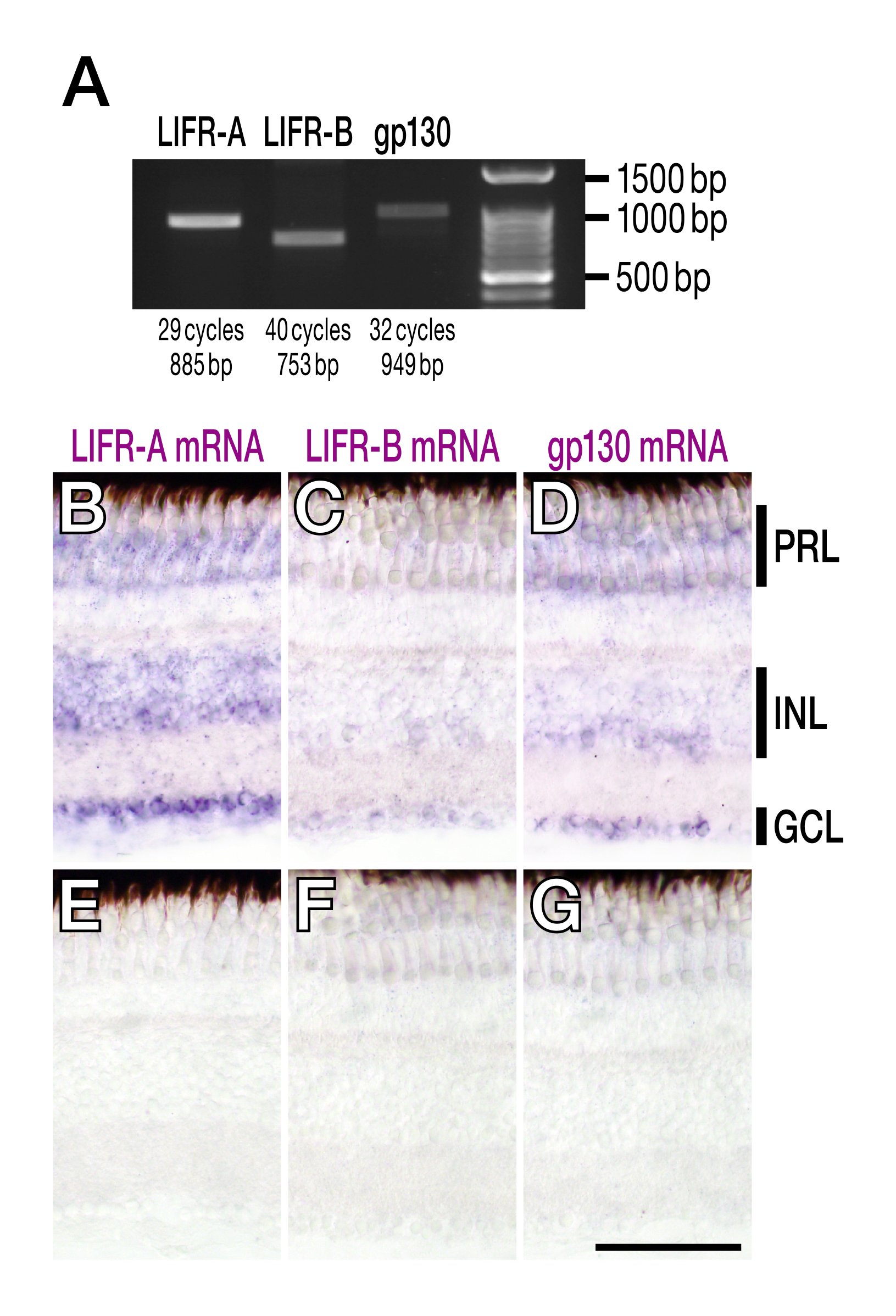

Supplement: Figure S2 — LIF receptor expression in the retina of adult zebrafish. (A) RT-PCR of two LIF receptors (LIFR-A and LIFR-B) and gp130 on uninjured retinal cells. All of these receptor genes are expressed in the zebrafish retina, although their expression levels vary greatly. (B–G) In situ hybridization experiments with LIF receptors. The localization of LIFR-A, LIFR-B, and gp130 mRNA is shown in (B), (C), and (D), respectively. All of the receptors are expressed in the GCL and other cell layers at the same levels as shown by RT-PCR (A). Sense probes yielded no specific staining (E–G). The scale bar in (G) is 50 µm. GCL: ganglion cell layer, INL: inner nuclear layer, PRL: photoreceptor layer. (TIF) [file pone.0106010.s002.tif]

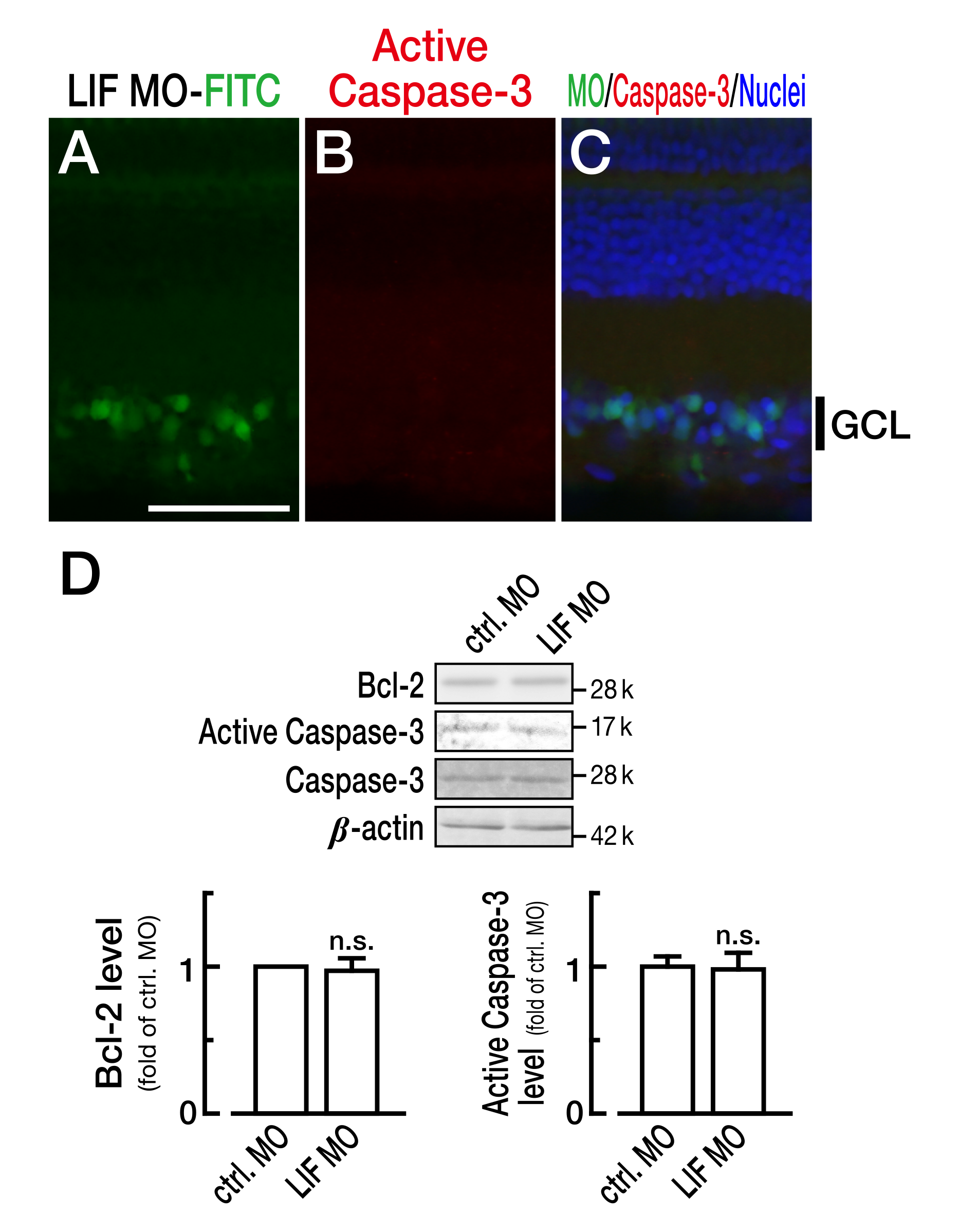

Supplement: Figure S3 — No changes in the expression of pro-apoptotic and anti-apoptotic factors after a LIF knockdown. (A–C) Immunohistochemical staining of active caspase-3, the effector molecule of apoptotic cell death, in LIF MO-treated retina at 10 dpi. Caspase-3 was not activated by LIF knockdown. (D) Changes in Bcl-2, the anti-apoptotic factor, and active caspase-3 levels in the control MO- and LIF MO-treated retina (10 dpi). Neither the level of Bcl-2 nor that of active caspase-3 was affected by LIF knockdown (n = 4; p = 0.88 and p = 0.89, respectively); n.s.: not significant. GCL: ganglion cell layer. (TIF) [file pone.0106010.s003.tif]

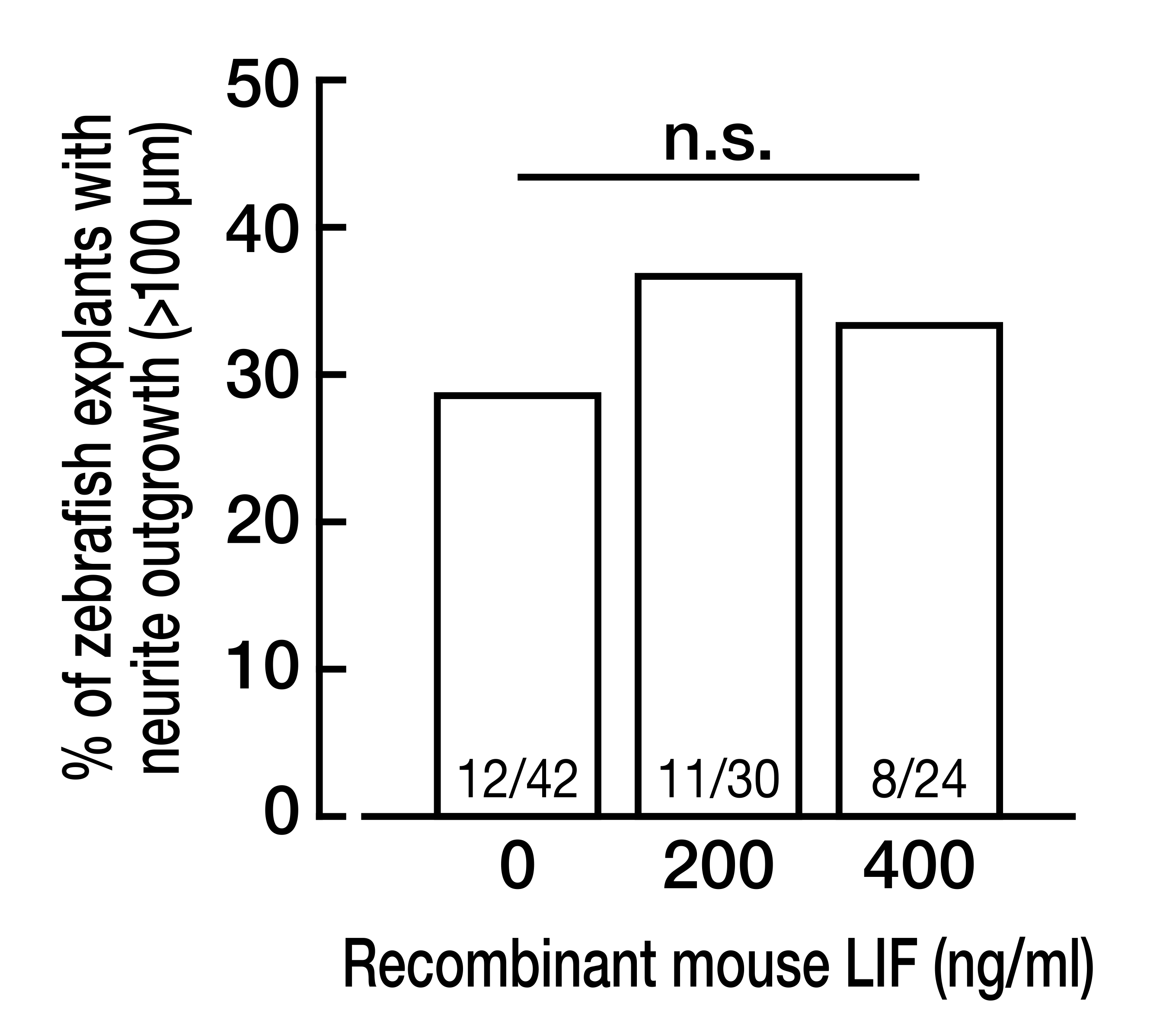

Supplement: Figure S4 — Addition of recombinant mouse LIF to zebrafish retinal explant culture. Supplementation with recombinant mouse LIF had no effect on the ratio of explants showing neurite outgrowth (p = 0.76); n.s.: not significant. (TIF) [file pone.0106010.s004.tif]
