# Supplementary material for: Upregulation of Leukemia Inhibitory Factor (LIF) during the Early Stage of Optic Nerve Regeneration in Zebrafish
Source: PLoS One. 2014 Aug 27;9(8):e106010. doi: 10.1371/journal.pone.0106010 (PMC4146584; doi:10.1371/journal.pone.0106010)
Supplement: Table S1 — Primers used for quantitative real-time PCR. (PDF) [file pone.0106010.s005.pdf]

Table S1. Primers used for quantitative real-time PCR

Gene Name	Accession #	Sequences (5′→ 3′)	Product size (bp)	Efficiency (%)
IL-6 (*il6*)	NM_001261449.1	Fw: GACATCCAGCACTTCCCTATCC<br>Rv: TTCCCTCTTTTCCTCCTGCTC	138	95.5
CNTF (*cntf*)	NM_001145632.1	Fw: GCGACTGGTGGGAGTTTTG<br>Rv: AGCACCTCTTCTTGTCCGTTG	150	94.7
LIF (*m17*)	NM_001079833.1	Fw: CAAGTCAAATTCAGAGCATACTTCG<br>Rv: TGAGCTTCAGACTTCGGTGAA	121	95.5
β-actin (*actb1*)	NM_131031.1	Fw: CGCAAATACTCCGTCTGGAT<br>Rv: GCACTTCCTGTGAACGATGG	120	95.2

Fw: forward, Rv: reverse
