# Supplementary material for: Upregulation of Leukemia Inhibitory Factor (LIF) during the Early Stage of Optic Nerve Regeneration in Zebrafish
Source: PLoS One. 2014 Aug 27;9(8):e106010. doi: 10.1371/journal.pone.0106010 (PMC4146584; doi:10.1371/journal.pone.0106010)
Supplement: Table S2 — Primers used for qualitative PCR and in situ hybridization probe. (PDF) [file pone.0106010.s006.pdf]

Table S2. Primers used for qualitative PCR and in situ hybridization probe

Gene Name	Accession #	Sequences (5´→ 3´)	Product size (bp)
LIF (*m17*)	NM_001079833.1	Fw: AGGAACATGCTCTGCCTGTC<br>Rv: AGCTGCTGAAGCCGAGTTAG	578
LIFR-A (*lifra*)	NM_001014306.1	Fw: GTGTCCGTGTTTGCTCTGTC<br>Rv: GTCTTGGGGCTCGTCTGG	885
LIFR-B (*lifrb*)	NM_001113732.1	Fw: TTAGACCAGCAAAAAGGCATC<br>Rv: CACAGCAATGGGAAGTGG	753
gp130 (*il6st*)	NM_001113504.1	Fw: TGCTGGAGTGGGTGAATG<br>Rv: TGATGGTTTGGGGTTTGG	949

Fw: forward, Rv: reverse
