# Supplementary material for: Upregulation of Leukemia Inhibitory Factor (LIF) during the Early Stage of Optic Nerve Regeneration in Zebrafish
Source: PLoS One. 2014 Aug 27;9(8):e106010. doi: 10.1371/journal.pone.0106010 (PMC4146584; doi:10.1371/journal.pone.0106010)
Supplement: Table S3 — Primary antibodies used in this study. (PDF) [file pone.0106010.s007.pdf]

**Table S3. Primary antibodies used in this study**

| Protein Name | Type | Supplier | Catalog # | Dilution |
|---|---|---|---|---|
| LIF | mouse monoclonal | Santa Cruz, CA, USA | sc-80159 | 1:100 (WB)<br>1:50 (IHC) |
| STAT3 | rabbit polyclonal | Santa Cruz | sc-7179 | 1:300<br>(WB, IHC) |
| phospho-STAT3 (pTyr$^{708}$) | mouse monoclonal | Medical & Biological Laboratories, Nagoya, Japan | D128-3 | 1:500 (WB)<br>1:300 (IHC) |
| Bcl-2 | rabbit polyclonal | Santa Cruz | sc-492 | 1:200 (WB) |
| active Caspase-3 | rabbit polyclonal | BD Biosciences, CA, USA | #559565 | 1:500 (WB)<br>1:300 (IHC) |
| β-actin | rabbit polyclonal | GeneTex, CA, USA | GTX16039 | 1:500 (WB) |
| βIII-tubulin (Tuj1) | rabbit monoclonal | Cell Signaling Technology, MA, USA | #5666 | 1:50 (IHC) |
| GAP-43 | goal polyclonal | Santa Cruz | sc-17790 | 1:300<br>(WB, IHC) |

WB: western blotting, IHC: immunohistochemistry
